# Expression and function of mechanosensitive ion channels in human valve interstitial cells

**DOI:** 10.1371/journal.pone.0240532

**Published:** 2020-10-15

**Authors:** Hessah Al-Shammari, Najma Latif, Padmini Sarathchandra, Ann McCormack, Eva A. Rog-Zielinska, Shahzad Raja, Peter Kohl, Magdi H. Yacoub, Rémi Peyronnet, Adrian H. Chester

**Affiliations:** 1 National Heart & Lung Institute, Imperial College London, London, United Kingdom; 2 Heart Science Centre, Magdi Yacoub Institute, Harefield, United Kingdom; 3 Institute for Experimental Cardiovascular Medicine, University Heart Center Freiburg, Bad Krozingen, and Faculty of Medicine, University of Freiburg, Freiburg, Germany; 4 Harefield Hospital, Royal Brompton & Harefield NHS Foundation Trust, Harefield, United Kingdom; Brigham and Women’s Hospital, Harvard Medical School, UNITED STATES

## Abstract

**Background:**

The ability of heart valve cells to respond to their mechanical environment represents a key mechanism by which the integrity and function of valve cusps is maintained. A number of different mechanotransduction pathways have been implicated in the response of valve cells to mechanical stimulation. In this study, we explore the expression pattern of several mechanosensitive ion channels (MSC) and their potential to mediate mechanosensitive responses of human valve interstitial cells (VIC).

**Methods:**

MSC presence and function were probed using the patch clamp technique. Protein abundance of key MSC was evaluated by Western blotting in isolated fibroblastic VIC (VIC_FB_) and in VIC differentiated towards myofibroblastic (VIC_MB_) or osteoblastic (VIC_OB_) phenotypes. Expression was compared in non-calcified and calcified human aortic valves. MSC contributions to stretch-induced collagen gene expression and to VIC migration were assessed by pharmacological inhibition of specific channels.

**Results:**

Two MSC types were recorded in VIC_FB_: potassium selective and cation non-selective channels. In keeping with functional data, the presence of both TREK-1 and Kir6.1 (potassium selective), as well as TRPM4, TRPV4 and TRPC6 (cationic non-selective) channels was confirmed in VIC at the protein level. Differentiation of VIC_FB_ into VIC_MB_ or VIC_OB_ phenotypes was associated with a lower expression of TREK-1 and Kir6.1, and a higher expression of TRPV4 and TRPC6. Differences in MSC expression were also seen in non-calcified *vs* calcified aortic valves where TREK-1, TRPM4 and TRPV4 expression were higher in calcified compared to control tissues. Cyclic stretch-induced expression of COL I mRNA in cultured VIC_FB_ was blocked by RN-9893, a selective inhibitor of TRPV4 channels while having no effect on the stretch-induced expression of COL III. VIC_FB_ migration was blocked with the non-specific MSC blocker streptomycin and by GSK417651A an inhibitor of TRPC6/3.

**Conclusion:**

Aortic VIC express a range of MSC that play a role in functional responses of these cells to mechanical stimulation. MSC expression levels differ in calcified and non-calcified valves in ways that are in part compatible with the change in expression seen between VIC phenotypes. These changes in MSC expression, and associated alterations in the ability of VIC to respond to their mechanical environment, may form novel targets for intervention during aortic valvulopathies.

## Introduction

Valve interstitial cells (VIC) play a key role in the maintenance and durability of heart valve cusps, owing to their ability to secrete extracellular matrix (ECM) proteins and to express enzymes responsible for ECM remodelling [1–3]. The processes mediated by VIC are, in part, influenced by mechanical cues to which these cells are exposed to during each cardiac cycle. The distending effect of the diastolic pressure exerts strain on the VIC as the cusps stretch during valve closure. Previous studies have shown that leaflet strain is approximately 23% in the radial direction [4], however due to the shielding effects of ECM, the strain experienced by cells is thought to be approximately two-thirds (e.g. ~15%) of the overall tissue strain [5], and sufficient to induce functional responses from the cells [3, 5].

Previous *in vitro* studies have confirmed that VIC are mechanosensitive, demonstrating functional responses to the application of stretch to cultured cells and tissues from a range of species, including human [6–9]. Observed responses include stretch-induced enhancement of proliferation, up-regulation of expression of genes encoding various ECM components, increased expression of matrix metalloproteinases, increased production of collagen, and heightened sensitivity to growth factors.

A variety of mechanosensitive proteins have been proposed to play a role in the transduction of stretch into biological responses by VIC, including integrin molecules and components of cell-cell junctions [10]. Mechanical stimuli applied to valve cusps is thought to be transmitted through the ECM to membrane mechanotransducers, and to the intracellular cytoskeleton. Integrin-mediated mechanotransduction, for example, can trigger the activation of various signalling pathways involving TGFß1, MAP kinases, Rho GTPases, and lead to elevation of cytoplasmic calcium levels and the generation of reactive oxygen species [10].

An alternative mechanotransduction pathway relies on the ability of cells to respond to mechanical stimuli *via* mechanosensitive ion channels (MSC). MSC convert signals such as in-plane membrane tension, membrane curvature, and tension within the cytoskeleton or the ECM into a biochemical or an electrochemical flux, which can affect cellular function [11, 12]. MSC can be subdivided into two main families, based on their ion selectivity: potassium-selective MSC (MSC_K_), such as TREK-1 and Kir6.1, and cation non-selective MSC (MSC_NS_), including transient receptor potential (TRP) channels. Members of these MSC families have been identified in a diverse range of cells and tissues, including the central and peripheral nervous system, myocardium, retina, kidney, lung, vascular endothelium and connective tissue [13]. Specifically, a role for TREK-1 has been widely described in the cardiovascular system [14], while mutations in the gene encoding Kir6.1 have been implicated in Cantu syndrome, a rare condition associated with bicuspid aortic valves, aortic stenosis and mitral regurgitation [15, 16]. TRPV4 is involved in regulating cellular responses required for valve morphogenesis, while TRPC6 and TRPM4 are both involved in a number of mechanotransduction pathways in the cardiovascular system and are two of the few TRP channels that are activated by membrane stretch [17].

The presence, expression patterns and potential functional roles of MSC in valve cells have not yet been comprehensively addressed, especially in valves from adult patients. The aim of this study is to characterise the expression of a number of key MSC in VIC (Table 1), and to determine how changes in VIC phenotype, that are associated with the onset and progression of calcific valve disease, affect MSC expression. Furthermore, the contribution of these channels to mechano-regulated functional responses of VIC is assessed with respect to the expression of collagen genes and the migration of VIC. The production of collagen is a key property of VIC that helps maintain the integrity and durability of the valve, with dysregulation of collagen production contributing to valve thickening. The migration of cells originating from the endothelium is a key step in valve formation and a source of new VIC *via* endothelial-to-mesenchymal transition [18, 19]. The overall goal of the study is to determine potential roles of MSC in valve mechanotransduction pathways in the response to physiological and pathophysiological changes in VIC phenotypes.

**Table 1:** Channel names, and gene names.

Channel	Full Name	Gene Name	Aliases
Kir6.1	Potassium inwardly rectifying channel, member 6.1	*KCNJ8*	uKATP-1
TREK-1	TWIK-related potassium channel, member 1 (TWIK standing for: tandem of two-pore potassium domains in a weak inwardly rectifying potassium channel)	*KCNK2*	K_2P_2.1
TRPM4	Transient receptor potential cation channel subfamily Melastatin, member 4	*TRPM4*	FLJ20041
TRPV4	Transient receptor potential cation channel subfamily Vanilloid, member 4	*TRPV4*	TRP12, VROAC
TRPC6	Transient receptor potential cation channel subfamily Canonical, member 6	*TRPC6*,	TRP6, FSGS2

## Materials and methods

Non-calcified aortic valves were collected from the Royal Brompton & Harefield Heart Valve Bank. Valves used here were classed as unsuitable for clinical use due to damage during harvesting/ transportation, the presence of early signs of calcification on valves, sinus wall or the coronary ostia, or issues with the medical history of the donor (cancer, recent infection, recent body tattooing). Donors were between 21 to 81 years of age (58% male, 42% female). Calcified valves were collected from patients undergoing aortic valve surgery at Harefield Hospital. This cohort comprised of patients who were between 56–85 years of age (78% male, 22% female). The collection and use of human samples were approved by the North London Research Ethics Committee (Ref: 10/H072418). All studies were performed in accordance with the ethical standards laid down in the 2013 Declaration of Helsinki. All donors, or their families, gave written informed consent prior to use of donated tissue.

### Cell isolation and culture

Human aortic valve leaflets were excised and washed in phosphate buffered saline (PBS). Valve leaflets were incubated in 0.15% w/v Type A collagenase (Roche Life Sciences, United States) and forcefully agitated for 10 minutes at 37°C to remove endothelial cells. The de-endothelialised valve tissue was washed again in PBS and digested for a further 3 hours to isolate VIC. The VIC suspension was centrifuged for 7 min at 700 × g, and the resulting pellet resuspended in Dulbecco’s Modified Eagle Medium (DMEM) containing 2% heat-inactivated foetal calf serum (FCS), 150 U/mL penicillin/streptomycin, 2 mM L-glutamine (all from Sigma, Gillingham, UK) and plated into tissue culture flasks which were maintained at 37°C under 5% CO_2_. When cells had become confluent and passaged, they were subsequently maintained in media without streptomycin or penicillin. In preliminary experiments we determined that the presence of streptomycin had no effect on the levels of expression of MSC (S1 Fig).

### Fibroblast and osteoblast VIC differentiation

In order to keep VIC in a fibroblast phenotype (VIC_FB_), they were maintained for a minimum of 2 weeks in streptomycin-free DMEM containing 2% FCS, 150 U/mL, 2 mM L-glutamine, 50 ng/mL insulin and 10 ng/mL fibroblast growth factor-2 (Sigma, Gillingham, UK) as previously described [20]. Myofibroblastic VIC (VIC_MB_) were obtained by culturing VIC_FB_ for 2 weeks in streptomycin-free DMEM containing 150 U/mL, 2 mM L-glutamine and 10% FCS, as previously described [21]. To differentiate cells into an osteoblastic VIC phenotype (VIC_OB_), cells were incubated for at least 3 weeks in media used for VIC_MB_, supplemented with 10 mM β-glycerophosphate, 50 μg/mL ascorbic acid, and 10 nM dexamethasone, as previously described [22]. The absence or presence of myofibroblast makers was assessed in VIC_FB_ and VIC_MB_ cell cultures, and cultures of VIC_OB_ were shown to express RUNX2 and osteopontin (see S2 Fig). All cells were used at a passage numbers no greater than six.

### RNA extraction, cDNA synthesis and quantitative PCR

RNA was extracted using the RNeasy mini kit (Qiagen, Crawley, UK) according to manufacturer’s protocols. Reverse transcription was carried out with the TaqMan Reverse Transcriptase Kit (Applied Biosystems, Birchwood, UK) according to manufacturer’s protocols. Each reaction was performed on 5 μg of total RNA. The resulting cDNA (100 ng/reaction) was used in quantitative PCR using the ABI Prism 7500 system (Applied Biosystems, Lincoln City, CA, USA). 18S ribosomal RNA was used to normalise expression levels of the genes of interest (housekeeping gene).

### Western blotting

Protein detection was performed in RIPA buffer (Sigma) lysed VIC. Total protein levels were quantified with a Pierce BCA protein assay (Thermo Scientific, Hemel Hempstead, UK). Protein lysates (7.5 μg) were electrophoretically separated under denaturing conditions on 10% Bis-Tris gels (Invitrogen, Renfrew, UK), and transferred on to nitrocellulose membranes (Hybond C, Amersham, UK). Specific MSC were detected using following antibodies: Kir6.1 (Bioss, London, UK), TREK-1, TRPM4, TRPV4, TRPC6 (Abcam, Cambridge, UK), followed by washing and incubation with secondary antibodies. Bands were visualized using enhanced chemiluminescence substrate and positivity was captured on Hyperfilm (GE Healthcare, Amersham, UK). Films were scanned and bands were quantified using the QuantityOne program (Biorad, Hercules, USA). Levels of protein expression were normalised to those of glyceraldehyde 3-phosphate dehydrogenase (GAPDH) (R&D Systems, Abingdon, UK).

### Immunohistochemistry

Non-calcified and calcified valves were fixed in 10% neutral buffered formalin for 24 hours (calcified valves were additionally decalcified in 10% formic acid for 2–3 days) and paraffin-embedded. Prior to immunoperoxidase staining, 5 μm thick paraffin wax sections of valves were dewaxed and rehydrated in water and washed in PBS for 5 minutes. Antigen retrieval was carried out by microwaving the slides in 0.1 M citrate buffer for 10 minutes and leaving them in the same buffer for further 20 minutes. The blocking for endogenous peroxidases was carried out using 0.3% hydrogen peroxide in PBS for 10 minutes. Sections were washed twice in PBS and blocked with 3% w/v bovine serum albumin in PBS for 30 minutes, and then incubated separately overnight with rabbit polyclonal antibodies against TREK-1 at 1:200 dilution (Antibodies-online, Aachen, Germany), rabbit polyclonal against Kir 6.1 at 1:50 dilution, mouse monoclonal against TRPM4 at 1:50 dilution, rabbit polyclonal against TRPV4 at 1:400 dilution, rabbit polyclonal against TRPC6 at 1:200 dilution (all from Abcam). Primary antibodies were omitted for negative controls. Specific staining was detected using biotinylated goat anti-mouse immunoglobulins for mouse antibodies and goat anti-rabbit immunoglobulins for rabbit antibodies (Vector Laboratories, Peterborough, UK). Sections were then washed 3 times in PBS and incubated with Avidin-Biotin Complex ABC for 30 minutes (Vector laboratories). Reactivity was detected using diaminobenzidine tetrahydrochloride (DAB tablets; 25 mg/mL) and hydrogen peroxide (0.01% w/v; Sigma). Sections were then counter stained with haematoxylin and viewed on a Zeiss Axioskop microscope. Photomicrographs were taken using Nikon DMX1200 camera.

### Patch clamp

Electrophysiological recordings were performed in cell-attached and inside-out modes, at room temperature, with an Axon 200B patch-clamp amplifier and a Digidata 1440A interface (Axon Instruments, Foster City, CA, USA). Current recordings were digitized at 10 kHz, low-pass filtered at 1 kHz, and analysed with pCLAMP10.3 (Axon Instruments) and ORIGIN9.1 (OrginLab Corporation, Northampton, MA, USA) software. The pipette medium contained: 150 mM NaCl, 5 mM KCl, 1 mM CaCl_2_, and 10 mM HEPES (pH 7.4 adjusted with NaOH), 10 mM tetraethylammonium, 5 mM 4-aminopyridine, and 10 μM glibenclamide, in order to minimize possible contamination of electrophysiological recordings by BK, Kv, or K_ATP_ currents. The bath medium contained: 155 mM KCl, 5 mM EGTA, 3 mM MgCl_2_, and 10 mM HEPES (pH 7.2 with KOH). At least 10 min before recordings, culture medium was removed and exchanged for the bath solution to wash-out streptomycin and to expose cells to the standard ionic conditions used to record MSC. Brief (255 ms) negative pressure pulses from 0 mmHg to -80 mmHg (in -10 mmHg increments) were applied to membrane patches *via* the recording electrode, using a pressure-clamp device (ALA High Speed Pressure Clamp-1 system; ALA Scientific, Farmingdale, USA).

### Cyclic mechanical stimulation of cultured cells

VIC_FB_ were stretched in culture, using a Flexercell FZ-4000T strain unit (Flexcell International Corporation, Burlington, USA). VIC were seeded at 5x10^4^ cells per well onto the centre of BioFlex 6-well culture plates, pre-coated with collagen type I and maintained in fibroblast medium not containing streptomycin. Cells were allowed to adhere (without stretch application) for 2 days in an incubator at 37°C with 5% CO_2_. Prior to being stretched, a visual check was made that cells had covered the deformable membrane in each well. Culture dish membranes were cyclically stretched equibiaxially by 15%, at 1 Hz for 24 hours. The amount of stretch applied reflects the level of strain experienced by cells under physiological conditions [4, 5]. VIC_FB_ cultured on BioFlex plates as described above, but not stretched served as the control group. In a subset of experiments, cells were pre-incubated with one of the following: 100 μM streptomycin (a blocker of MSC_NS_ [23, 24]), 1 μM spadin (TREK-1 blocker, [25, 26]), 0.5 μM RN-9893 (TRPV4 blocker, [27]), or 1 μM GSK417651A (TRPC6/3 blocker, [28, 29]). (Streptomycin was purchased from Sigma; GSK417651A was purchased from Tebu-Bio Ltd, Peterborough, UK; Spadin and RN-9893 were purchased from Tocris, Abingdon, UK).

### Migration assay

VIC migration was assessed by making a scratch in a monolayer of cells using a 200 μL pipette tip, as previously described [30]. Cells were maintained in fibroblast media not containing streptomycin. The distance between the edges of the scratch was measured immediately after it had been made, and again after 72 hours, to calculate the percentage of gap closure. The concentration of FCS in the medium was maintained at 0.4% throughout to counter cell proliferation.

### Statistics

Data are represented by box-and-whisker plots as median ± the 25^th^ and 75^th^ percentile (the box) and ± the minimum and maximum value (whiskers), using Prism 7 (GraphPad Software, San Diego, CA, USA). Statistical significance of any differences between groups of data was assessed using a one-way ANOVA followed a Tukey’s multiple comparisons test for experiments with three or more groups, or a one-tailed, non-paired t-test for comparisons between two groups. *P* values of less than 0.05 were considered to indicate a significant difference between the means. Values for *p <0*.*05* are indicated on the figures and shown in the figure legends, together with the number of individuals from which cells or tissue were obtained. Exact p values for statistically significant differences are given the S1 Table.

## Results

### VIC display MSC_K_ and MSC_NS_ activity

**Fig 1:**
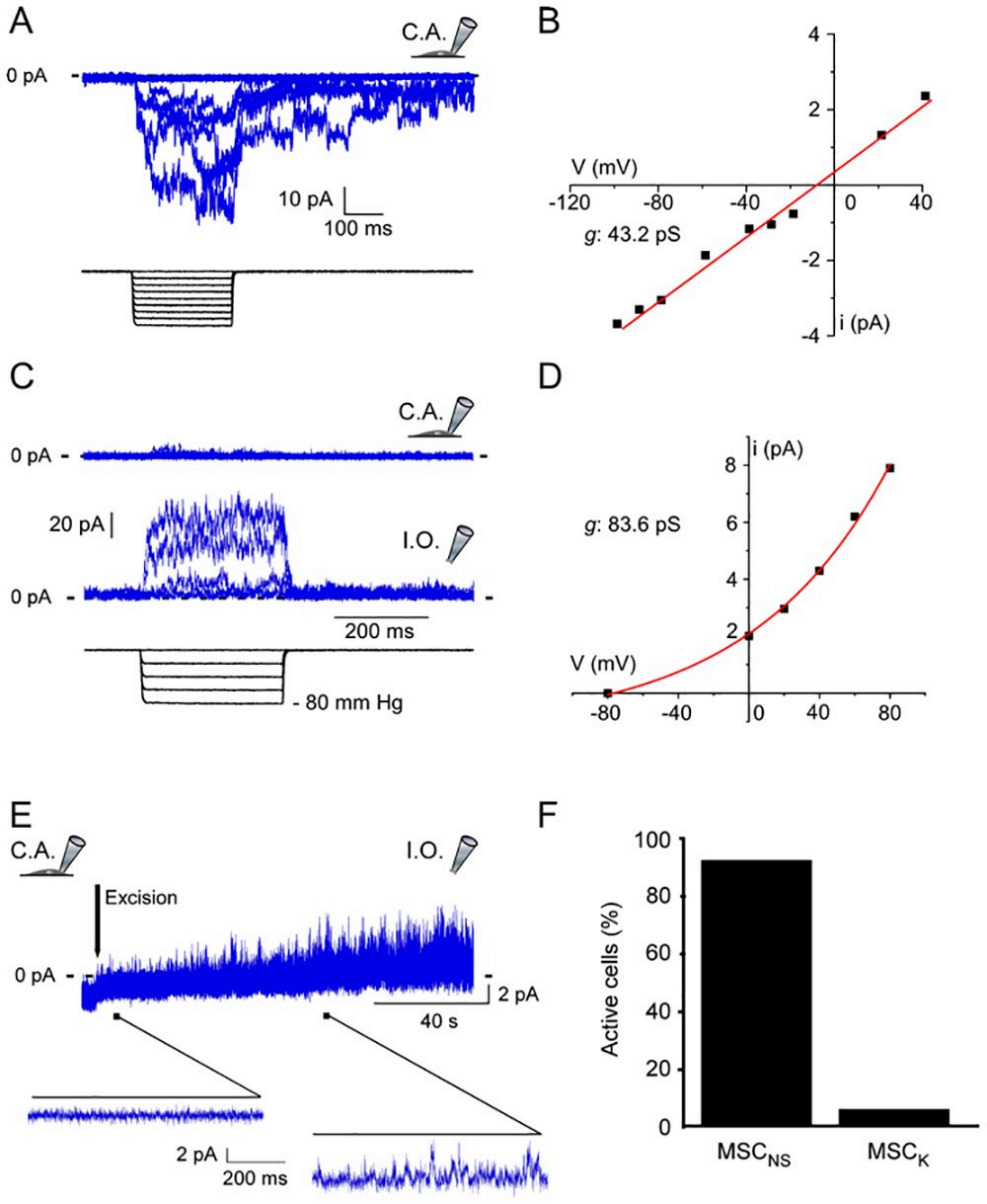
Mechanosensitive ion channel activity in human aortic valve interstitial cells. A: Representative recording of a cation non-selective mechanosensitive channel (MSC_NS_) in cell-attached patch configuration (C.A.), activated in response to 255 ms long negative pressure pulses, from 0 to −80 mm Hg, applied in increments of −10 mm Hg, at a holding potential of -80 mV. Each trace shows the mechanically-induced changes in membrane current in response to applied pressure. B: Current-voltage relation obtained from MSC_NS_ single channel activity at a pressure of -20 mm Hg in the cell-attached configuration for a representative cell. C: Representative recording of a potassium-selective MSC (MSC_K_) in cell-attached and in inside-out configurations, in response to 500 ms long negative pressure pulses, from 0 to -80 mmHg, applied in increments of -20 mm Hg, at a holding potential of 0 mV. D: Current-voltage relation obtained from MSC_K_ single channel activity at a pressure of -20 mm Hg in the inside-out configuration for a representative cell. E: The typical progressive activation of MSC_K_ following patch excision. F: Percentage of cells in which the two types of MSC were recorded, MSC_NS_ = 156 cells (from 8 patients), MSC_K_ = 64 cells (from 5 patients).

In order to assess the presence of MSC at the plasma membrane, negative pressure pulses, delivered through the patch pipette were applied to VIC_FB_ in primary culture. Cells displayed two types of MSC activity. The more abundant activity, recorded at -80 mV (Fig 1A), has a near-linear current-voltage relation for single channel openings and a reversal potential at about -10 mV, suggesting the presence of MSC_NS_ (Fig 1B). The other activity is recorded at 0 mV with no or very small mechanically activatable currents in the cell-attached configuration, while MSC activity is unmasked by switching to inside-out mode (Fig 1C). The current-voltage relation of this MSC shows a reversal potential of -80 mV, suggesting presence of MSC_K_ (Fig 1D). MSC_K_ activity appears within the first minute after patch excision from the cell (Fig 1E). MSC_NS_ were detected in most cells tested (92% out of 156 cells), while MSC_K_ were rare (5% out of 64 excised patches, Fig 1F).

### Effect of VIC phenotype on MSC expression

**Fig 2:**
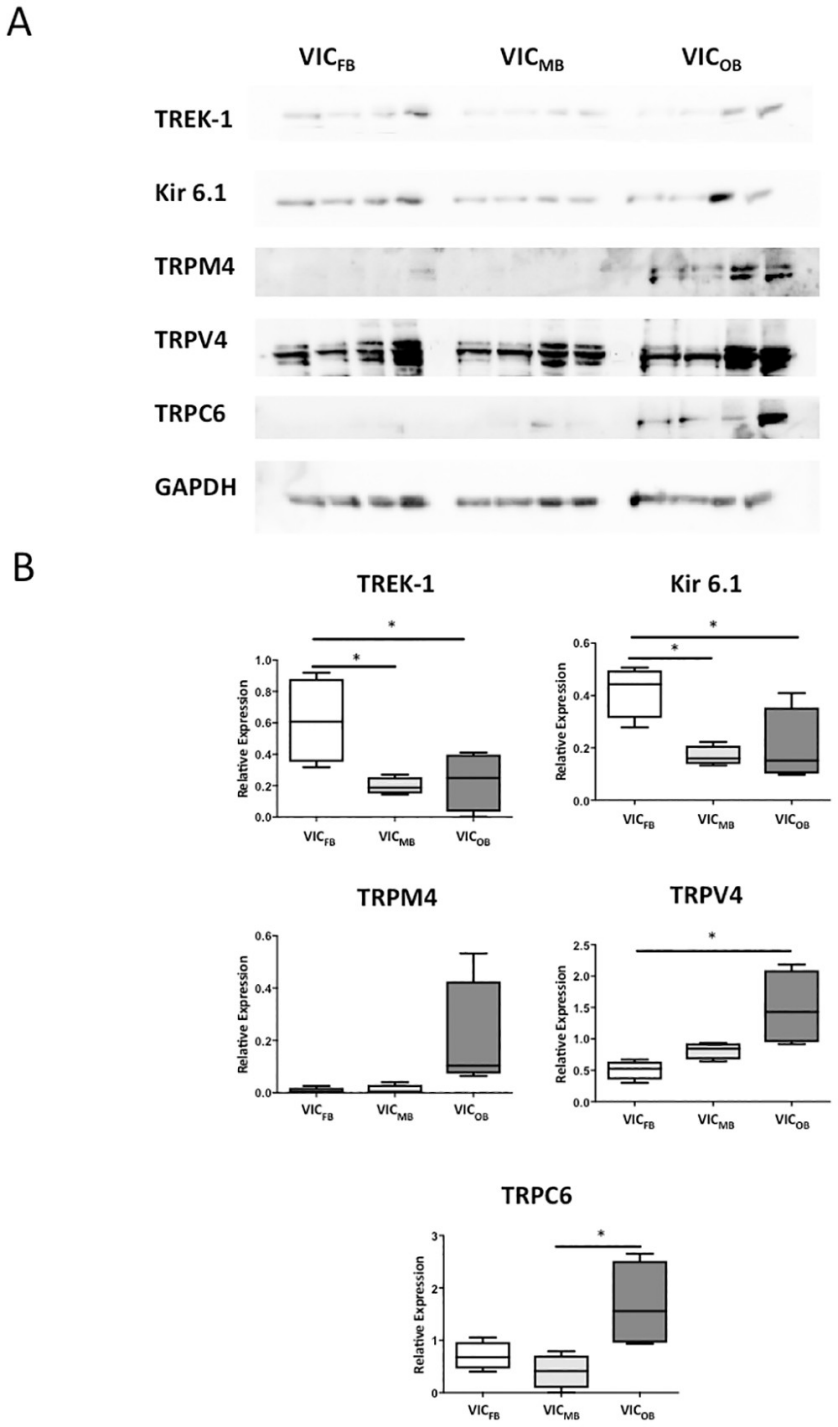
Protein levels of mechanosensitive ion channels in phenotypically different VIC populations. A: Western blots showing expression of MSC in cultures of VIC_FB_, VIC_MB_, and VIC_OB_. B: Quantification of relative expression of individual channel proteins in cells of each phenotype, normalized to GAPDH levels (n = 4 patients; * = p<0.05).

In order to determine which channels could be responsible for the mechanically-inducible ion currents observed in the path clamp experiments, MSC protein expression was investigated in three common VIC phenotypes. Protein expression of MSC_K_ (such as TREK-1 and Kir6.1) and MSC_NS_ (TRPM4, TRPV4, TRPC6) was analysed in VIC isolated from non-calcified valves, and maintained in culture conditions to drive their phenotypes towards VIC_FB_, VIC_MB_ or VIC_OB_. Expression of TREK-1 and Kir6.1 was higher in VIC_FB_ compared to VIC_MB_ and VIC_OB_, while protein levels of TRPV4 and TRPC6 were higher in VIC_OB_ compared to VIC_FB_ and VIC_MB_ respectively. Expression of TRPM4 was not statistically different between different cell phenotypes (Fig 2).

### Expression of MSC in non-calcified and calcified cusp tissue

**Fig 3:**
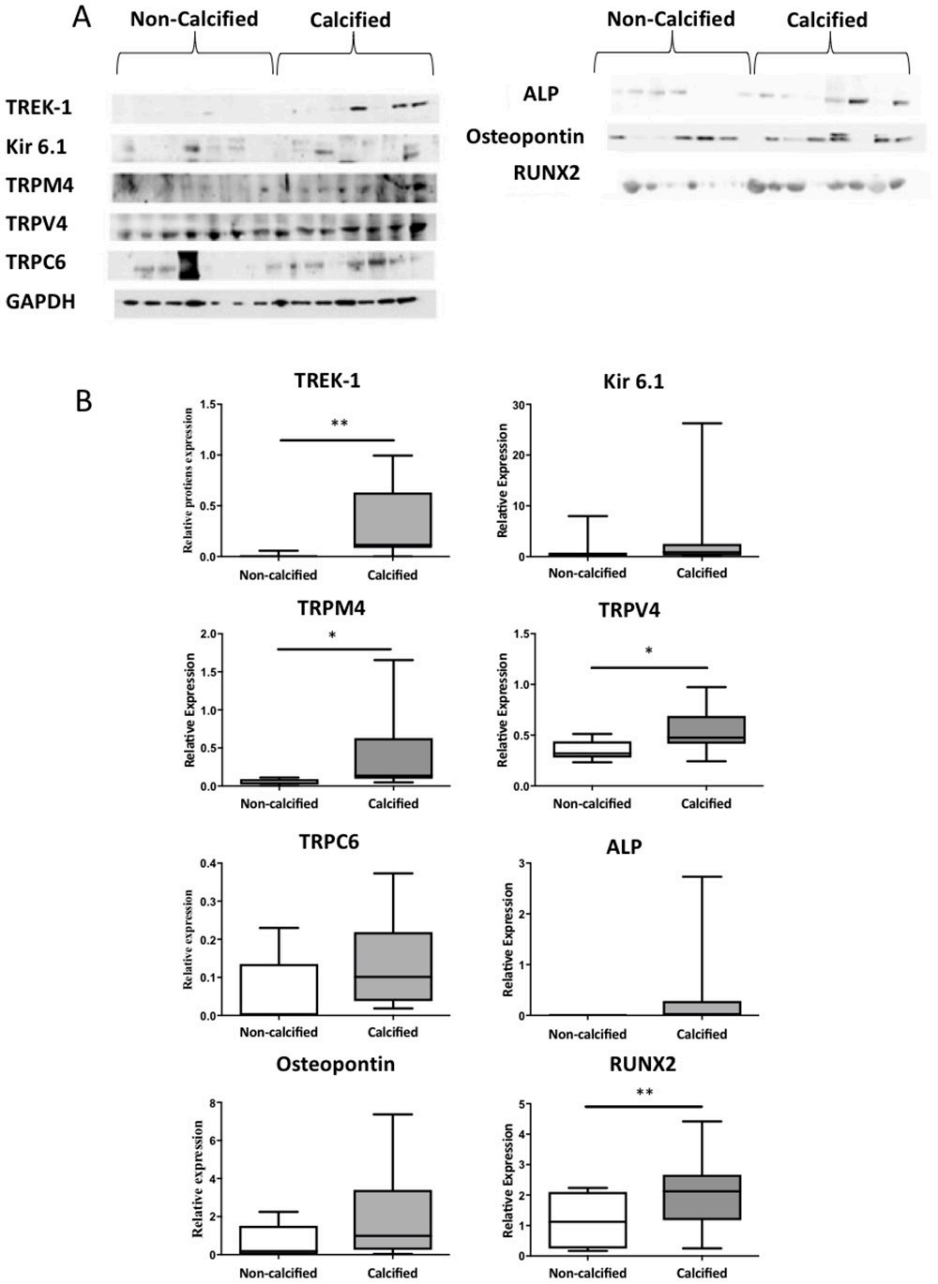
Protein expression of mechanosensitive ion channels in non-calcified and calcified aortic valve tissue from patients. A: Western blots showing expression of various MSC, and of markers of calcification in cusp tissue from calcified and non-calcified valves. B: Quantification of relative expression of individual channel proteins and calcification markers in non-calcified and calcified valves (n = 6 patients; * = p<0.05, ** = p<0.01).

**Fig 4:**
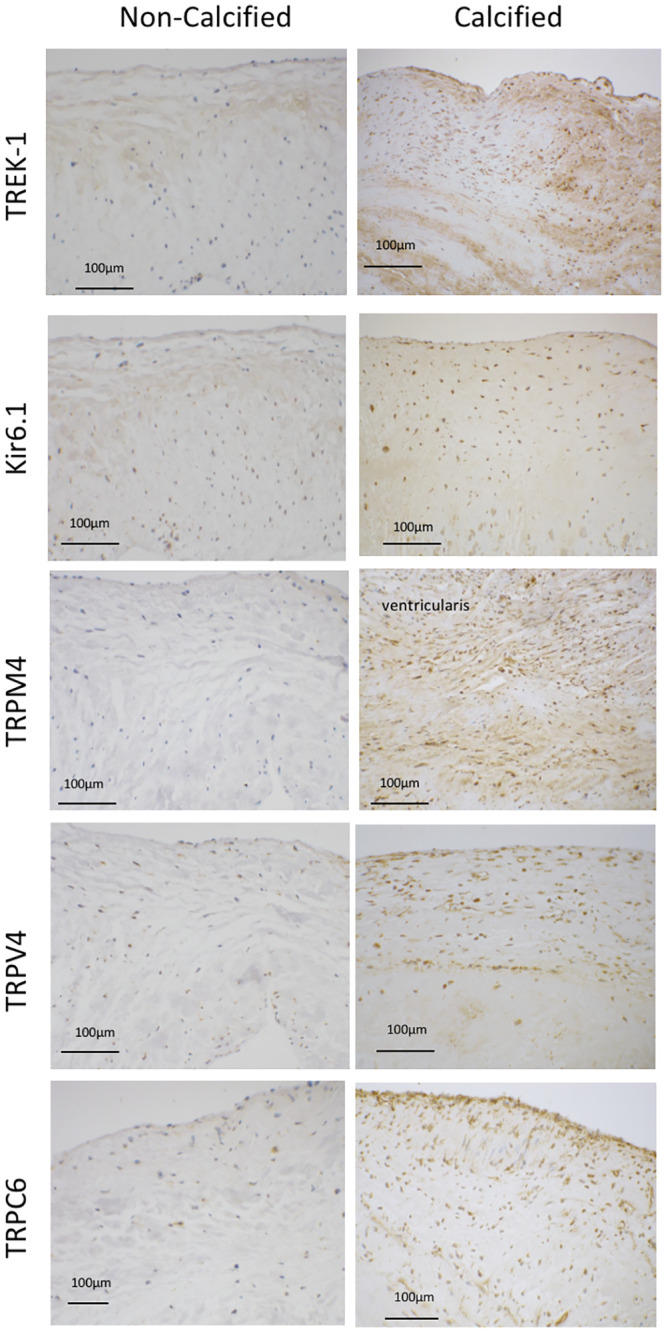
Representative images of immunohistochemical localisation of mechanosensitive ion channel in non-calcified and calcified aortic valve cusp tissue. Tissue sections were stained with antibodies against MSC_K_ TREK-1 and Kir6.1, and against MSC_NS_ TRPM4, TRPV4, and TRPC6. Signal (brown staining) from each antibody was assessed on sections from 8–10 individual valves. The sections are orientated with the ventricularis at the top of each section.

The differences observed in MSC expression in cultured cells suggests that channel expression varies between physiological and pathophysiological VIC phenotypes. To investigate whether these differences were observed in valve tissue, MSC expression was investigated in intact tissue samples. Protein levels of the MSC_K_ TREK-1 and of the MSC_NS_ TRPM4 and TRPV4 were higher in cusps from calcified compared to non-calcified valves, but there was no difference in the expression of Kir6.1 and TRPC6 (Fig 3). The expression of the osteoblast transcription factor RUNX2 was higher in calcified valves, while levels of osteopontin and alkaline phosphatase were unchanged (Fig 3). Immunohistochemical staining revealed that in non-calcified valves, expression of all MSC was constrained to a small proportion of VIC, and to endothelial cells on the aortic and ventricular surfaces of the cusp. In contrast, in calcified tissue, TREK-1 and Kir6.1, as well as TRPM4, TRPV4, and TRPC6 channels were strongly expressed by most VIC (Fig 4).

### Role of MSC in stretch-mediated increase in collagen expression by VIC_FB_

**Fig 5:**
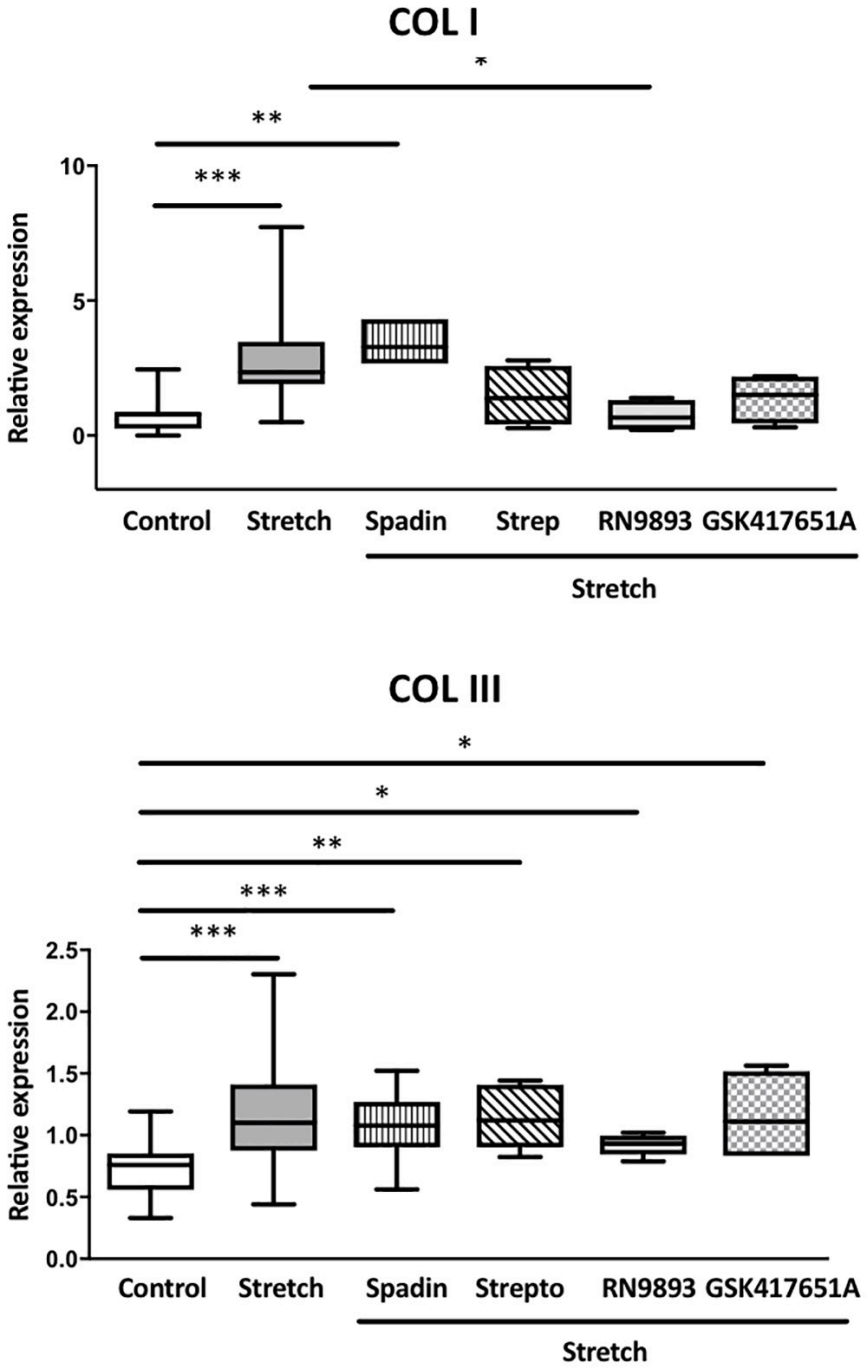
Role of TREK-1, TRPV4 and TRPC6/3 channels in stretch-induced changes in collagen mRNA expression in VIC_FB_. Changes in mRNA expression (relative to 18S) for COL I and COL III in VIC_FB_ under static conditions (white bars), after cyclic stretch (grey bars), and after stretch in the presence of either the TREK-1 channel inhibition by spadin, the general MSC_NS_ blocker streptomycin, the TRPV4 inhibitor RN-9893 or the TRPC6/3 inhibitor GSK417651A (hatched bars) after 24 h of stretch. Gene expression was measured by RT-PCR and normalized to the housekeeping gene (n = 4 and 6 patients for COL I and III respectively; * = p<0.05, ** = p<0.01, *** = p<0.001).

In an attempt to establish if any of the MSC expressed in VIC_FB_ play a role in the response of cells to mechanical stimulation, we investigated the effect of inhibitors of TREK-1, TRPV4 and TRPC6/3 on the increased expression of collagen in response to stretch. Cyclically stretched VIC_FB_ showed an increase in mRNA expression of type I and III collagen (COL I and COL III) after 24 hours compared to static cells (Fig 5), but not for elastin (data not shown). The stretch-induced increase in COL I mRNA expression was inhibited by the TRPV4 channel inhibitor RN-9398 and or, but not by streptomycin a general inhibitor of MSC_NS_. No significant effects from the TRPC6/3 inhibitor GSK417651A and the TREK-1 inhibitor spadin were observed (Fig 5). The increase in COL III mRNA expression induced by stretch was not affected by any of the MSC channel inhibitors.

### Role of MSC in the migration of VIC_FB_

**Fig 6:**
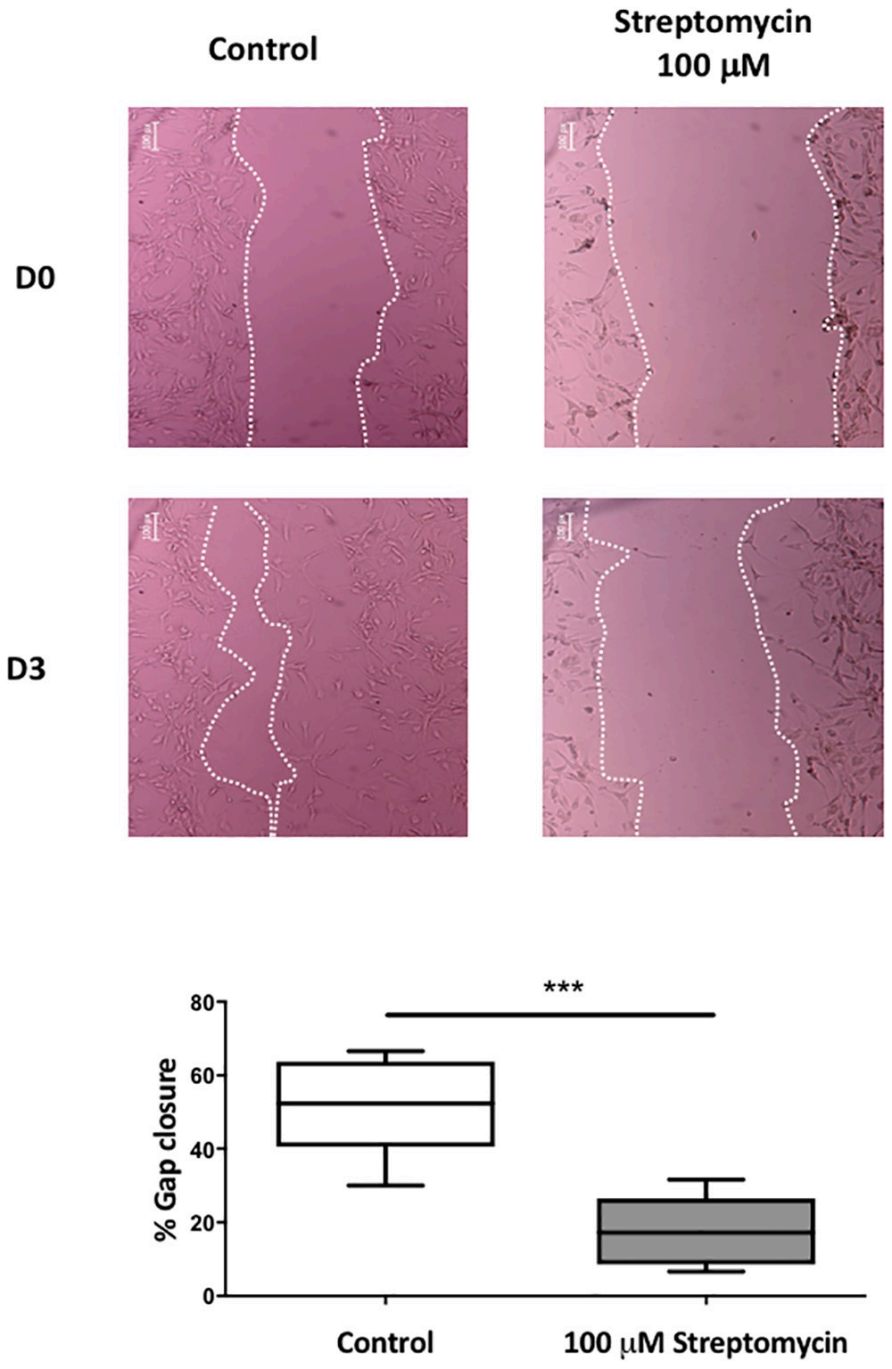
Effect of streptomycin on VIC_FB_ migration in a cell-culture scratch assay. Phase-contrast microscopy image of scratch assays, showing VIC_FB_ and scratch edge on Day-0 (top row) and Day-3 (bottom row) in the absence (control) and presence of 100 μM streptomycin. Scratch edge in each panel is indicated by the white dotted-line. Quantification of migration (day-3) in the control and streptomycin treated cells is shown the lower panels (n = 6 patients; *** = p<0.001).

**Fig 7:**
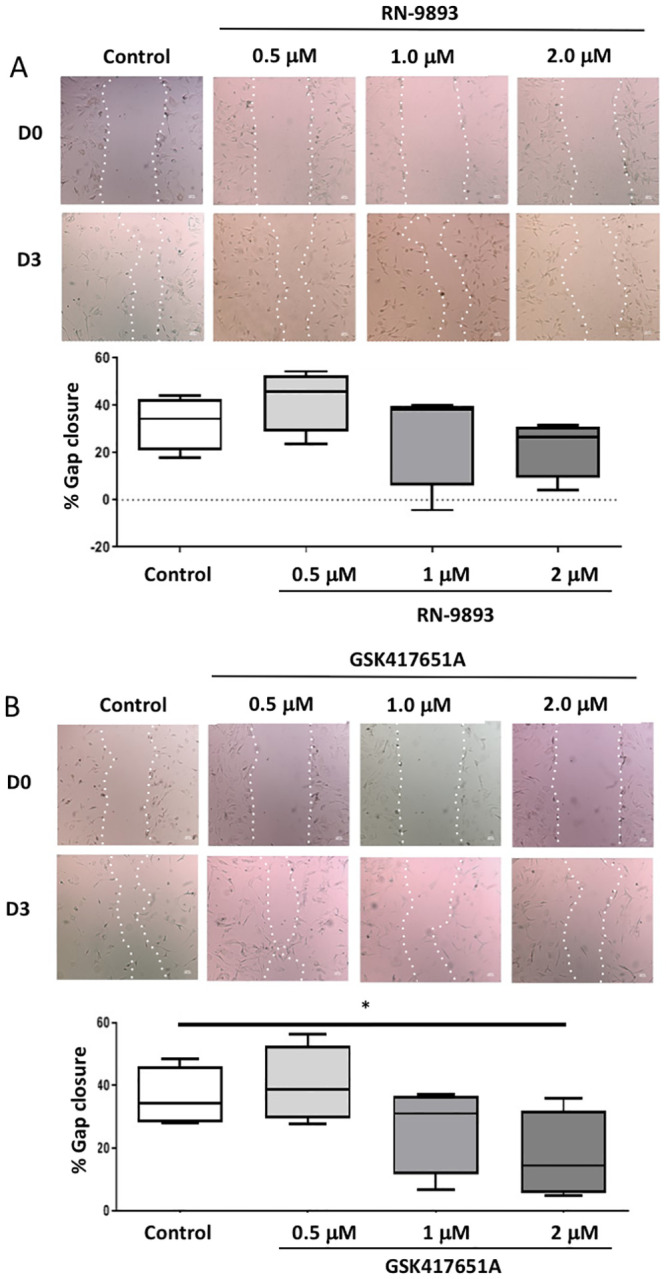
Effect of inhibitors of TRPV4 and TRPC6/3 on VIC_FB_ migration in a cell-culture scratch assay. Phase-contrast microscopy image of scratch assays, showing **VIC**_**FB**_ and scratch edge at Day-0 (top row) and Day-3 (bottom row) in the absence (control) and presence of different concentrations of (A) the TRPV4 inhibitor RN-9883 and (B) the TRPC6/3 inhibitor GSK417651A. Scratch edge in each panel is indicated by the white dotted-line. Quantification of migration (day 3) as a function of different concentrations of RN-9883 and GSK417651A is shown in the lower panels of A and B, respectively (n = 6 patients; * = p<0.05).

We also investigated the role of MSC in cell migration, a process that generates contractile and protrusive forces, causing cells to lengthen and shorten as they move across a substrate. Over a 72-hour period, migration of VIC_FB_ into the cell-free area of a scratch test assay reduced the distance between the two edges by 52.2 ± 13.9%. Gap closure was reduced to 17.5 ± 9.7% in the presence of 100 μM streptomycin (Fig 6). Migration of VIC_FB_ into the wounded area was also inhibited by the 2 μM TRPC6/3 inhibitor GSK417651A, but not by the TRPV4 inhibitor RN-9883 (Fig 7).

## Discussion

This study demonstrates the presence of two groups of MSC with distinct biophysical properties in human aortic VIC: MSC_K_ and MSC_NS_. The expression patterns of MSC change as VIC differentiate into pathophysiological cell phenotypes, and during the calcification process in human valve samples. MSC_NS_ further appear to play a role in VIC ECM component transcription in response to cyclic stretch, and in migratory activity.

The most frequently observed electrophysiological signature activity of MSC (present in ~90% of cells tested) was characteristic for MSC_NS_, while MSC_K_ activity was less frequently observed (in 5% of excised patches tested—as MSC_K_ activity is enhanced by excision of membrane patches from the cell—a well-known feature of TREK and TRAAK channels [31, 32]). Of note, the difference in MSC_NS_ *vs* MSC_K_ exists even though both sets of recordings assessed similarly sized membrane areas.

The presence of specific MSC_NS_ and MSC_K_ was confirmed by protein expression analysis of TREK-1, Kir6.1, as well as TRPM4, TRPV4, and TRPC6. While MSC_K_ were detected as trans-plasma membrane current pathways in a small fraction of cells only, protein levels detected in Western blots were significant. This suggests that MSC_K_ currents are either strongly inhibited (and, hence, not detectable even if present), or/and that a large fraction of the protein is located inside the cells (e.g. on endomembranes, where activity is not detectable with the patch clamp technique). In contrast, MSC_NS_ protein levels were low in Western blots, however observed single channel conductance is large, suggesting that only a few channels are required for significant ion fluxes. MSC expression appears to be influenced by phenotypic changes in VIC that occur during differentiation of VIC_FB_ into VIC_MB_ or VIC_OB_ *in vitro*, as well as during the process of valve calcification *in vivo*. In histological sections, MSC expression was also seen on endothelial cells on both sides of the valve. While this study has focused on the role of MSC in VIC, further studies are warranted to examine the role of these channels in valve endothelial cell function.

The influence of stretch on VIC function has been widely studied *in vitro*, illustrating the ability of these cells to secrete ECM proteins in response to mechanical cues, and thus providing a mechanism by which VIC may help to maintain the integrity and durability of valve cusps [6, 7, 33]. The mechanosensitivity of VIC is also evident *in vivo* by changes that occur in the ECM of semilunar valves after birth, as they adapt to difference in the haemodymanic environment between the foetal and neonatal circulation [34]. Collagen re-modelling has also been seen in the mitral valve of the maternal heart during pregnancy, in response to increased mitral valve strain [35]. Furthermore, valve adaptation to altered mechanics is seen following the Ross procedure (when a pulmonary valve is placed into aortic position) as pulmonary valves transition towards an aortic valve phenotype [36, 37].

The predominant cells in healthy valves are VIC_FB_ [38]. Under pathological conditions, VIC_FB_ become activated and differentiate into VIC_MB_. Eventually, as evidenced by the expression of bone markers, VIC_MB_ become VIC_OB_. TRPV4 and TRPC6 channels were upregulated in VIC_OB_ compared to VIC_FB_ and VIC_MB_ respectively, while there was a reduction in the expression TREK-1 and Kir6.1 channels in VIC_OB_ and VIC_MB_ compared to VIC_FB_. The expression TRPM4 and TRPV4, but not TRPC6, was upregulated in calcified valves, compared to non-calcified controls. In contrast to the reduced expression of TREK-1 and Kir6.1 in VIC_OB_ in culture, the expression of TREK-1, but not of Kir6.1, was higher in calcified valve tissue, compared to non-calcified tissue. RUNX2 expression was higher in calcified compared to non-calcified valves. Of note, while non-calcified valves were free of visible signs of calcification, and patients had no clinical history of cardiovascular disease, some expression of RUNX2 was observed in these samples. The expression of RUNX2 in non-calcified valve has previously been observed [39], but due to the age of some of these donors, the possibility exists that the expression of RUNX2 may reflect signs of initial activation of calcific pathways in these patients.

The changes in expression of MSC seen in pathological VIC phenotypes *in vitro* and in calcified tissue are suggestive of the functional relevance of changes in mechanical properties of VIC during the progression of valvular disease. It is known that, as valve calcification advances, cusps become progressively stiffer [40]. This increase in stiffness is associated with increased production of collagen, an effect that MSC appear to contribute to. The stretch-induced change seen in the expression of COL I was blocked by an inhibitor of TRPV4 channels. The identity of the MSC linked to the increased expression of COL III mRNA in response to stretch remains undetermined. Of note, the variability of the results corresponding to the stretch condition is higher compared to the static condition. This could be due to cell intrinsic properties, variability in adhesion for example, leading to inhomogeneity in the deformation applied due to cells being localised around the edge of the well where stretch is much less controlled compared to cells directly over the loading post.

It is possible that changes in the expression of MSC in calcified valves are part of a compensatory mechanism of adaptation, perhaps reducing the strain experienced by VIC in ECM-stiffened valve cusps. Additional studies are required to ascertain the contribution, if any, of MSC to the onset and progression of valve thickening and dysfunction.

MSC are also sensitive to forces generated by the cells. As cells migrate, forces and deformations affect the cell membrane when cells contract and elongate during movement [41]. This process is important for valve cells. During development, VIC are derived through the process of endothelial-to-mesenchymal transition (EMT), whereby endothelial cells that comprise the endocardial cushions differentiate into migratory cells that contribute to formation of valve cusps. There is evidence that this is a dynamic process by which the VIC population is continuously replenished from valve endothelial cells undergoing EMT and migrating into the valve cusp [42]. Pathological stimuli can promote EMT, potentially giving rise to VIC that become calcifying cells [19]. In our study, migration of VIC was blocked by the non-specific MSC_NS_ inhibitor streptomycin (used at 100 μM which is above concentrations required to robustly block cation nonselective stretch-activated channels (usually 30–50 mM)) [23], as well as by specific inhibitors of TRPC6/3 channels. TRP channels are known to play a key role in regulating the migration of cancer and endothelial cells, such as in tumour vascularisation [43]. We propose that they are also involved in VIC migration. It is not possible to derive from the current data how MSC channel inhibitors are able to reduce migratory activity of VIC_FB_, but inhibition of Ca^2+^ entry into the cells *via* MSC_NS_ channels could interfere with the contractile machinery and impede the motile capacity of the cell.

The inclusion of antibiotics is common in standard cell culture media and the presence of streptomycin at a concentration of 170 μM (1%) during the cell isolation and growth to the first passage, had no significant effect on the expression of MSC_K_ and MSC_NS_ (see S1 Fig). The effects of streptomycin on MSC_NS_ are reversible, permitting the isolation and initial growth of cells with antibiotics, prior to the use of antibiotic-free media for experiments to examine the activity and function of MSC_NS_. As a note of caution, keeping MSC_NS_ inhibited by streptomycin during the long-term culture of cells using standard culture conditions might have effects on cells. While the effects of streptomycin effects on MSC_NS_ are reversible, keeping the channels inhibited for long periods of time might have substantial effects on mechano-transduction processes involving MSC_NS_.

Overall, our study confirms the presence of various, electrophysiologically active MSC in VIC, and their contribution to functionally relevant responses in human valve cells. The presence of specific MSC was confirmed, both at mRNA and protein levels. The expression of these channels is affected by differentiation of VIC_FB_ into VIC_MB_ and VIC_OB_, which match some of the pathological changes observed in calcified valves. The use of pharmacological inhibitors has provided additional insight into contributions of MSC to collagen secretion in response to stretch, and to VIC migration. More definitive experiments are limited by the lack of selective inhibitors for other potential MSC. Additional experiments using molecular probes to silence or knock-down specific channels may provide further detail on the contribution of individual channels to functional responses of VIC to mechanical stimulation; these will be the focus of further research.

The precise contribution of these channels to valve function *in vivo* remains to be determined. Our findings add to the understanding of mechanisms by which VIC respond to their mechanical environment. In the long run, targeting MSC may offer new means of regulating heart valve adaptation and remodelling in response to alterations in their mechanical environment.

## Supporting information

S1 Fig The effect of streptomycin on the expression of MSC_K_ and MSC_NS_. Quantification of Western blots showing the relative expression of TREK-1, TRPV4 and TRPC6 channels in VIC grown in Fibroblast media in the presence and absence of 170 μM (1%) streptomycin for 2 weeks. (n = 6 patients; p>0.05).
(TIF)

S2 Fig Phenotypic characterisation of VIC_FB_, VIC_MB_ and VIC_OB_. Following differentiation of VIC_FB_ into VIC_M_B and VIC_OB_, the expression of phenotypic makers was assessed by western blotting for α-smooth muscle cell actin (α-SMA), transgelin (SM22), smooth muscle myosin heavy chain (SMM-HC), myocardin-related transcription factor-A (MTRFA), vimentin, calponin, myocardin, Runt-related transcription factor 2 (RUNX2), osteopontin and the house keeping protein GAPDH. The relative expression of each protein was calculated and is shown in the box and whisker plots in the lower part of the figure. Expression of α-SMA, SM22, SMM-HC, vimentin and myocardin were all significantly upregulated in VIC_MB_ compared to VIC_FB_. The osteoblast markers RUNX2, osteopontin were upregulated in VIC_OB_ compared to VIC_FB_ and VIC_MB_, α-SMA, SM22, MRTFA and calponin all remained significantly upregulated in VIC_OB_ compared to VIC_FB_. Myocardin and SMM-HC expression was similar to that in VIC_OB_ and VIC_FB_, but reduced compared to VIC_MB_ (n = 4 patients; * = p<0.05, *** = p<0.001).
(TIF)

S1 Table P values for statistically significant differences. (DOCX)

S1 Raw images (ZIP)
