# Supplementary figures and images for: Expression and function of mechanosensitive ion channels in human valve interstitial cells
Source: PLoS One. 2020 Oct 15;15(10):e0240532. doi: 10.1371/journal.pone.0240532 (PMC7561104; doi:10.1371/journal.pone.0240532)

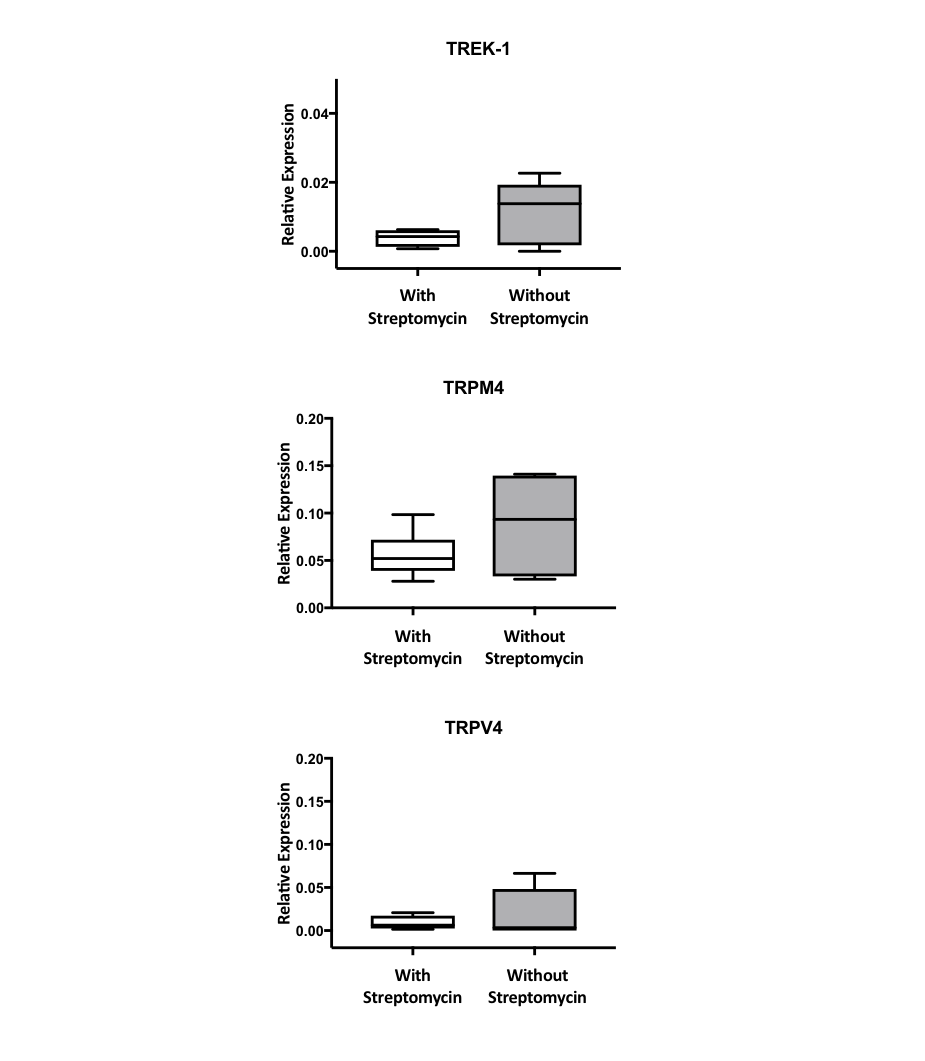

Supplement: S1 Fig — Quantification of Western blots showing the relative expression of TREK-1, TRPV4 and TRPC6 channels in VIC grown in Fibroblast media in the presence and absence of 170 μM (1%) streptomycin for 2 weeks. (n = 6 patients; p>0.05). (TIF) [file pone.0240532.s001.tif]

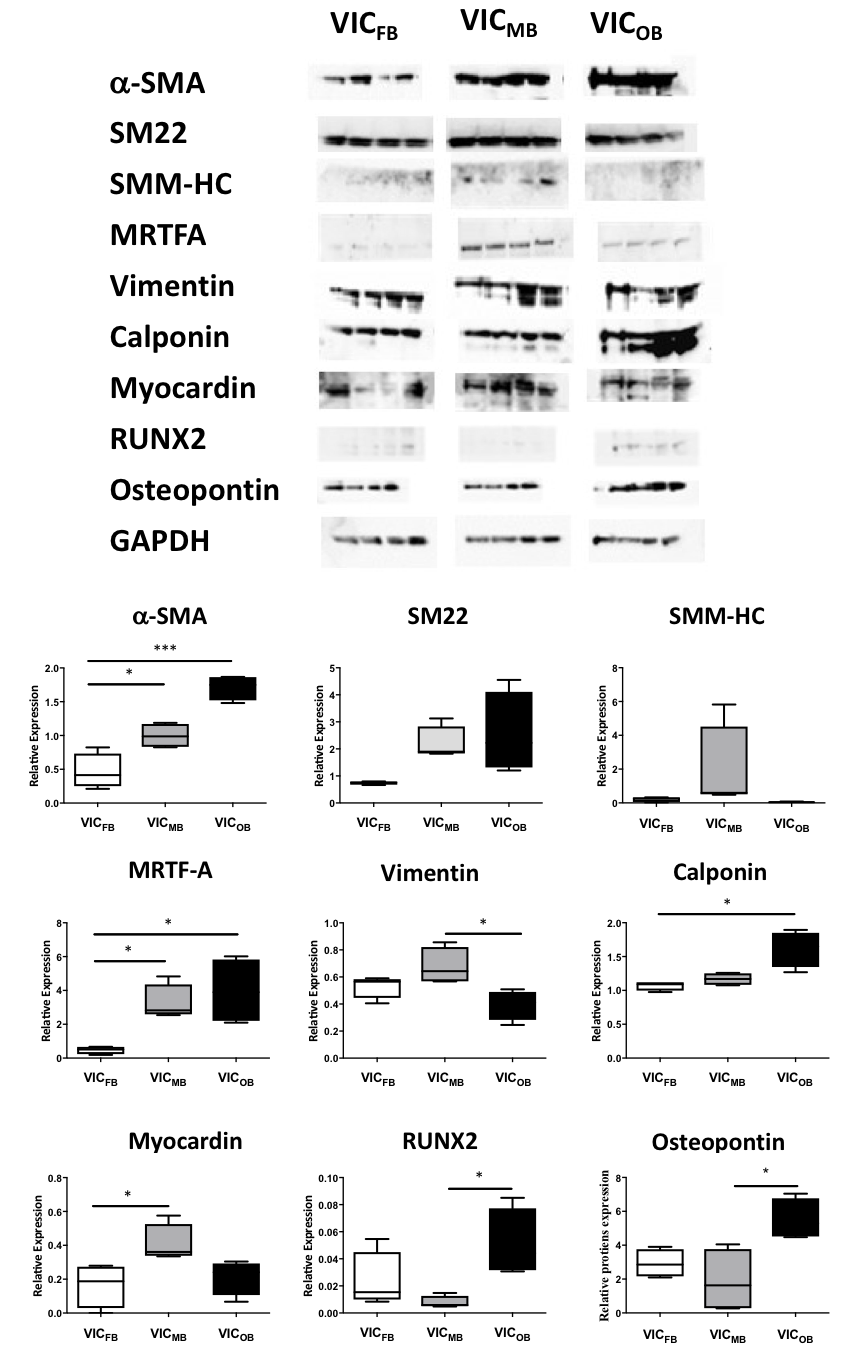

Supplement: S2 Fig — Following differentiation of VICFB into VICMB and VICOB, the expression of phenotypic makers was assessed by western blotting for α-smooth muscle cell actin (α-SMA), transgelin (SM22), smooth muscle myosin heavy chain (SMM-HC), myocardin-related transcription factor-A (MTRFA), vimentin, calponin, myocardin, Runt-related transcription factor 2 (RUNX2), osteopontin and the house keeping protein GAPDH. The relative expression of each protein was calculated and is shown in the box and whisker plots in the lower part of the figure. Expression of α-SMA, SM22, SMM-HC, vimentin and myocardin were all significantly upregulated in VICMB compared to VICFB. The osteoblast markers RUNX2, osteopontin were upregulated in VICOB compared to VICFB and VICMB, α-SMA, SM22, MRTFA and calponin all remained significantly upregulated in VICOB compared to VICFB. Myocardin and SMM-HC expression was similar to that in VICOB and VICFB, but reduced compared to VICMB (n = 4 patients; * = p<0.05, *** = p<0.001). (TIF) [file pone.0240532.s002.tif]
