# Supplementary material for: Expression and function of mechanosensitive ion channels in human valve interstitial cells
Source: PLoS One. 2020 Oct 15;15(10):e0240532. doi: 10.1371/journal.pone.0240532 (PMC7561104; doi:10.1371/journal.pone.0240532)
Supplement: S1 Table — (DOCX) [file pone.0240532.s003.docx]

**S1 Table. P values for statistically significant differences.**

| **Figure** | **Data Sets Compared** | **P value** |
| --- | --- | --- |
| Figure 2 |  |  |
| TREK-1 | VIC_FB_ - VIC_MB_ | 0.027 |
| TREK-1 | VIC_FB_ - VIC_OB_ | 0.039 |
| Kir 6.1 | VIC_FB_ - VIC_MB_ | 0.014 |
| Kir 6.1 | VIC_FB_ - VIC_OB_ | 0.029 |
| TRPV4 | VIC_FB_ - VIC_OB_ | 0.016 |
| TRPC6 | VIC_MB_ - VIC_OB_ | 0.028 |
|  |  |  |
| Figure 3 |  |  |
| TREK-1 | Non-calcified - Calcified | 0.002 |
| TRPM4 | Non-calcified - Calcified | 0.035 |
| TRPV4 | Non-calcified - Calcified | 0.036 |
| RUNX2 | Non-calcified - Calcified | 0.002 |
|  |  |  |
| Figure 5 |  |  |
| COL I | Control - Stretch | <0.001 |
| COL I | Control - Sapdin + Stretch | 0.001 |
| COL I | Stretch - RN9893 + Stretch | 0.031 |
| COL III | Control - Stretch | <0.001 |
| COL III | Control - Sapdin + Stretch | <0.001 |
| COL III | Control - Streptomycin + Stretch | 0.002 |
| COL III | Control – RN9893 + Stretch | 0.003 |
| COL III | Control – GSK417651A + Stretch | 0.002 |
|  |  |  |
| Figure 6 | Control - Streptomycin | <0.001 |
|  |  |  |
| Figure 7 | Control - 2µM GSK417651A | 0.039 |
|  |  |  |
| Figure S1 |  |  |
| α-SMA | VIC_FB_ - VIC_MB_ | 0.016 |
| α-SMA | VIC_FB_ - VIC_OB_ | <0.001 |
| MRTF-A | VIC_FB_ - VIC_MB_ | 0.033 |
| MRTF-A | VIC_FB_ - VIC_OB_ | 0.010 |
| Vimentin | VIC_MB_ - VIC_OB_ | 0.015 |
| Calponin | VIC_FB_ - VIC_OB_ | 0.023 |
| Myocardin | VIC_FB_ - VIC_MB_ | 0.036 |
| RUNX2 | VIC_MB_ - VIC_OB_ | 0.025 |
| Osteopontin | VIC_MB_ - VIC_OB_ | 0.012 |
|  |  |  |
