# Supplementary material for: Expression and function of mechanosensitive ion channels in human valve interstitial cells
Source: PLoS One. 2020 Oct 15;15(10):e0240532. doi: 10.1371/journal.pone.0240532 (PMC7561104; doi:10.1371/journal.pone.0240532)

Expression and function of mechanosensitive ion channels in human valve interstitial cells

Hessah Al-Shammari[1], Najma Latif [1,2], Padmini Sarathchandra[1], Ann McCormack[2], Eva A. Rog-Zielinska[3], Shahzad Raja[4], Peter Kohl[3], Magdi H. Yacoub[1,2], Rémi Peyronnet[2,3,‡] and Adrian H. Chester[1,2,‡,*]

Original Western Blots

Figure 2
Kir 6.1

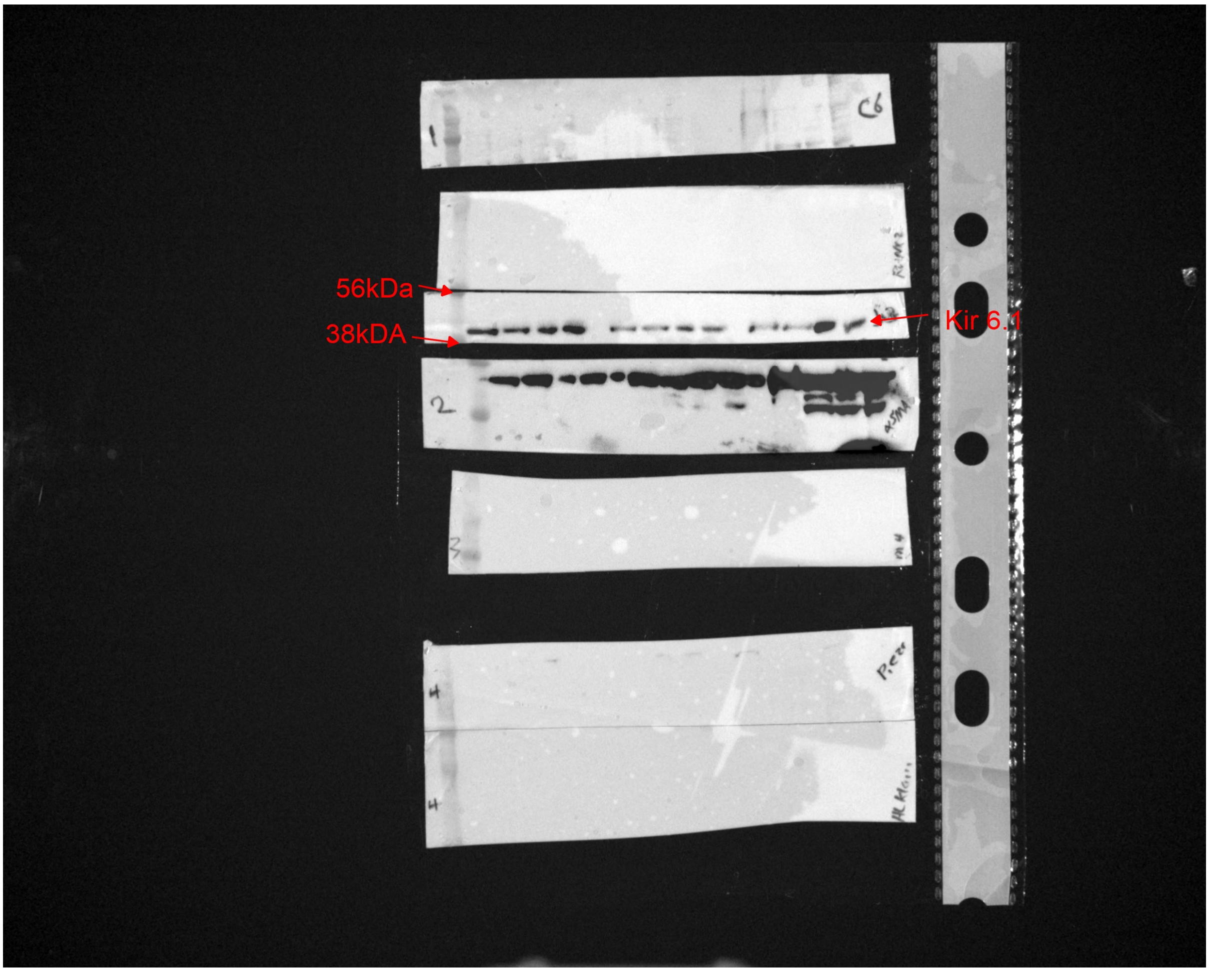

Figure 2
TRPC6 & TRPM4

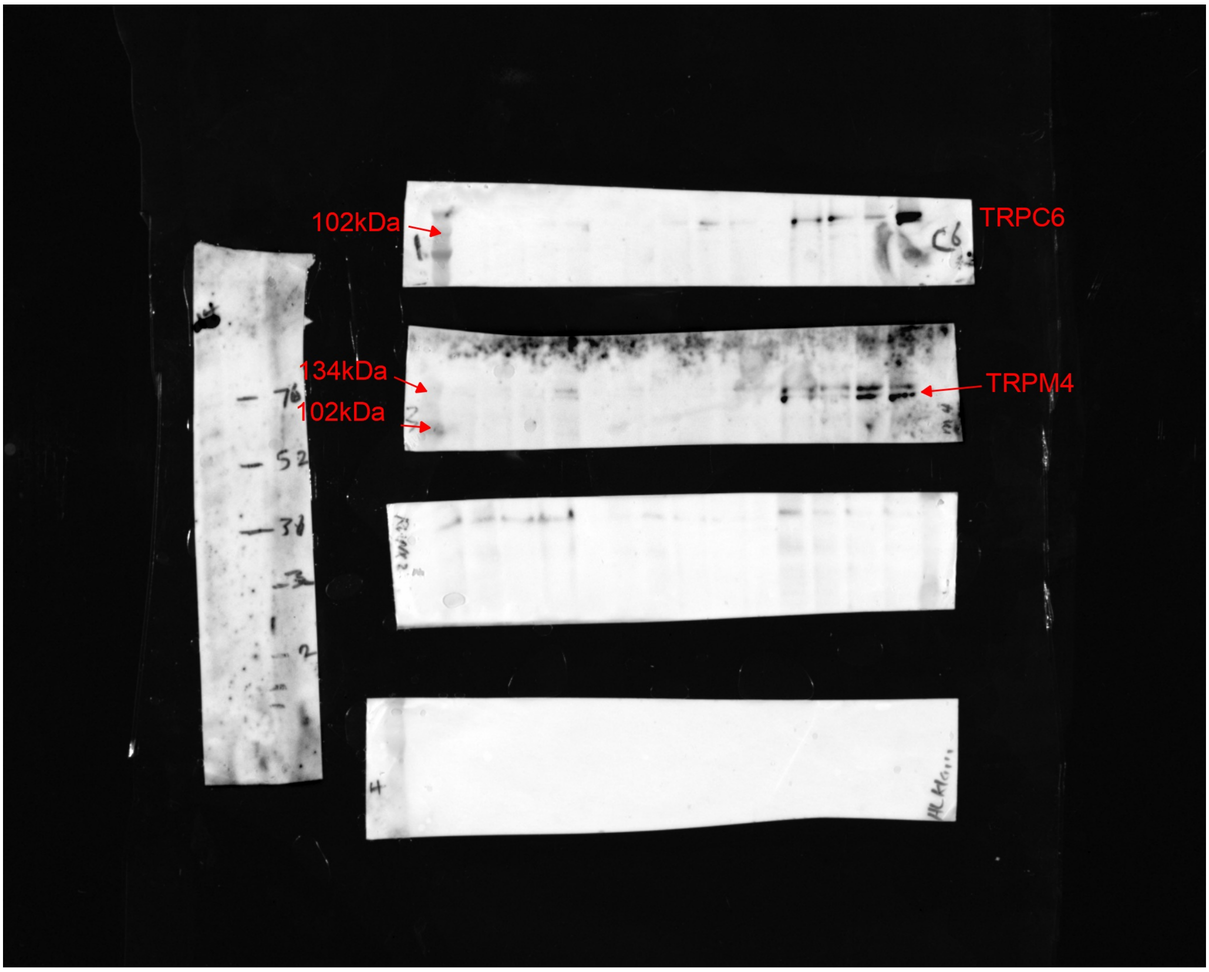

Figure 2
TRPV4

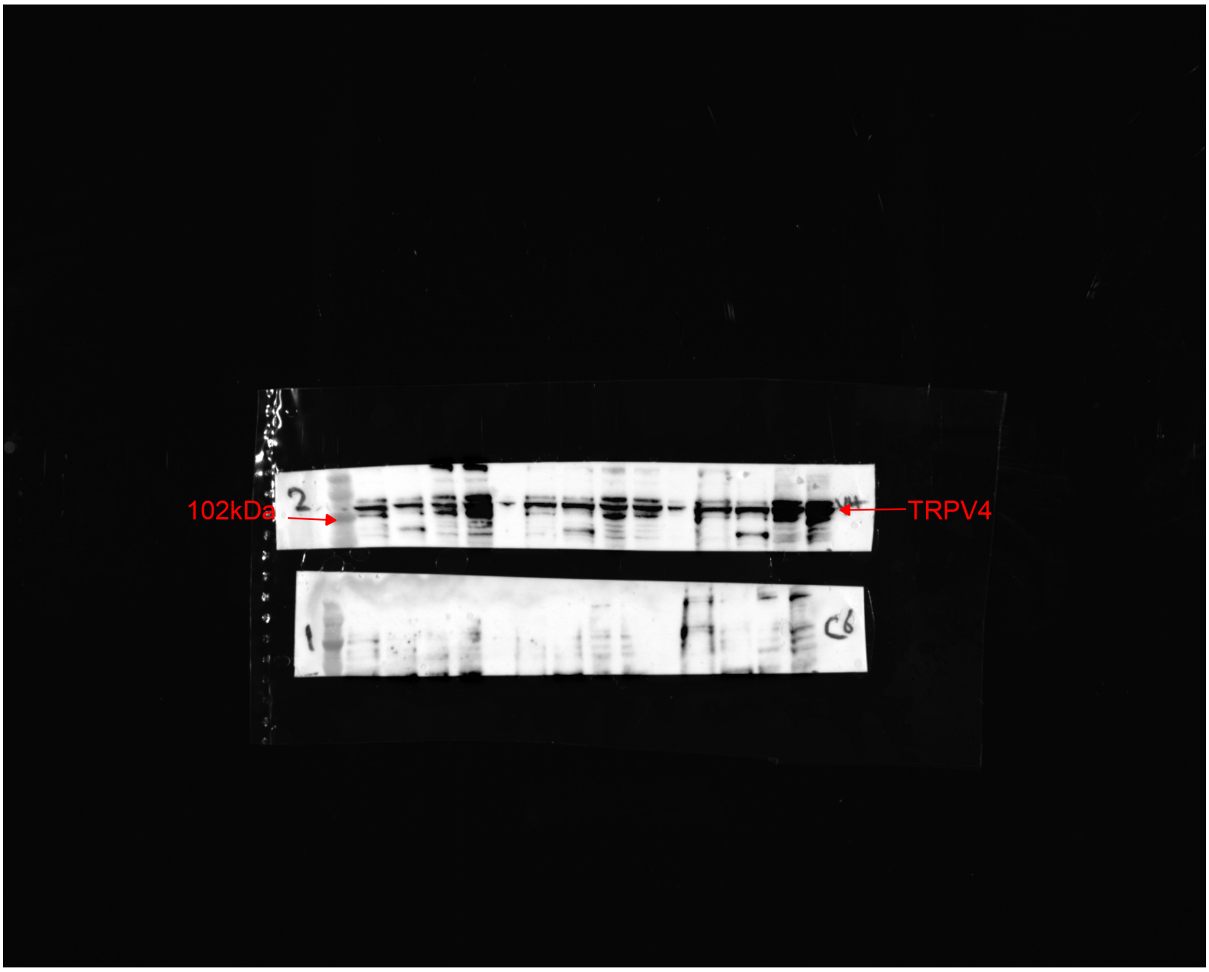

Figure 2
TREK1 & GAPDH

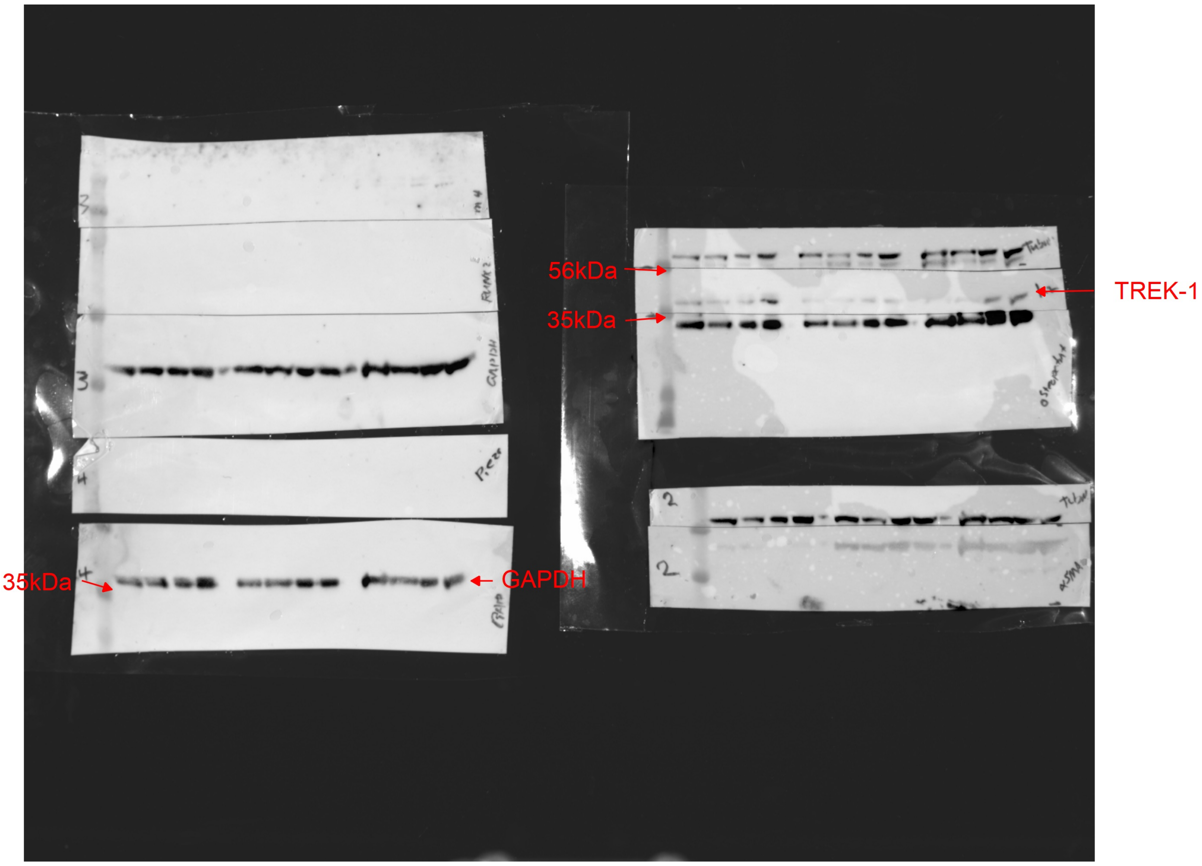

Figure 3
ALP & Kir 6.1

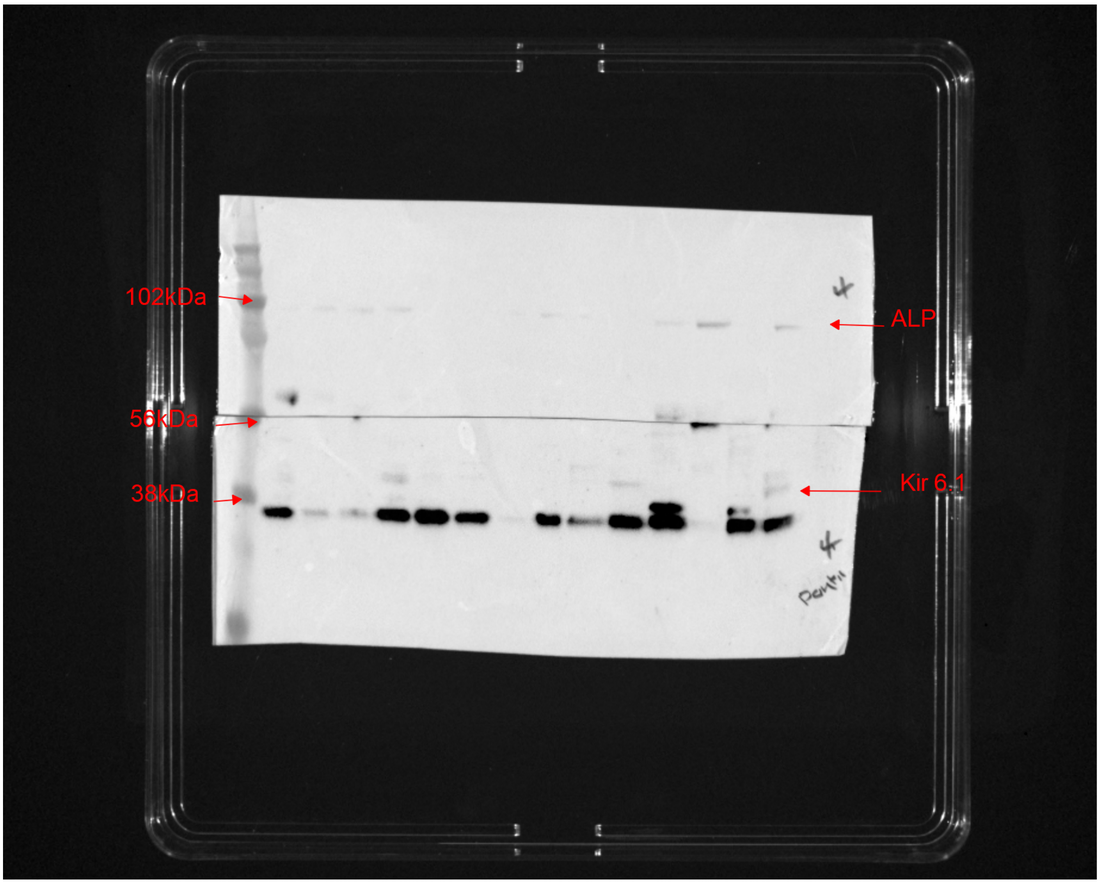

Figure 3
TRPC6 & TREK1

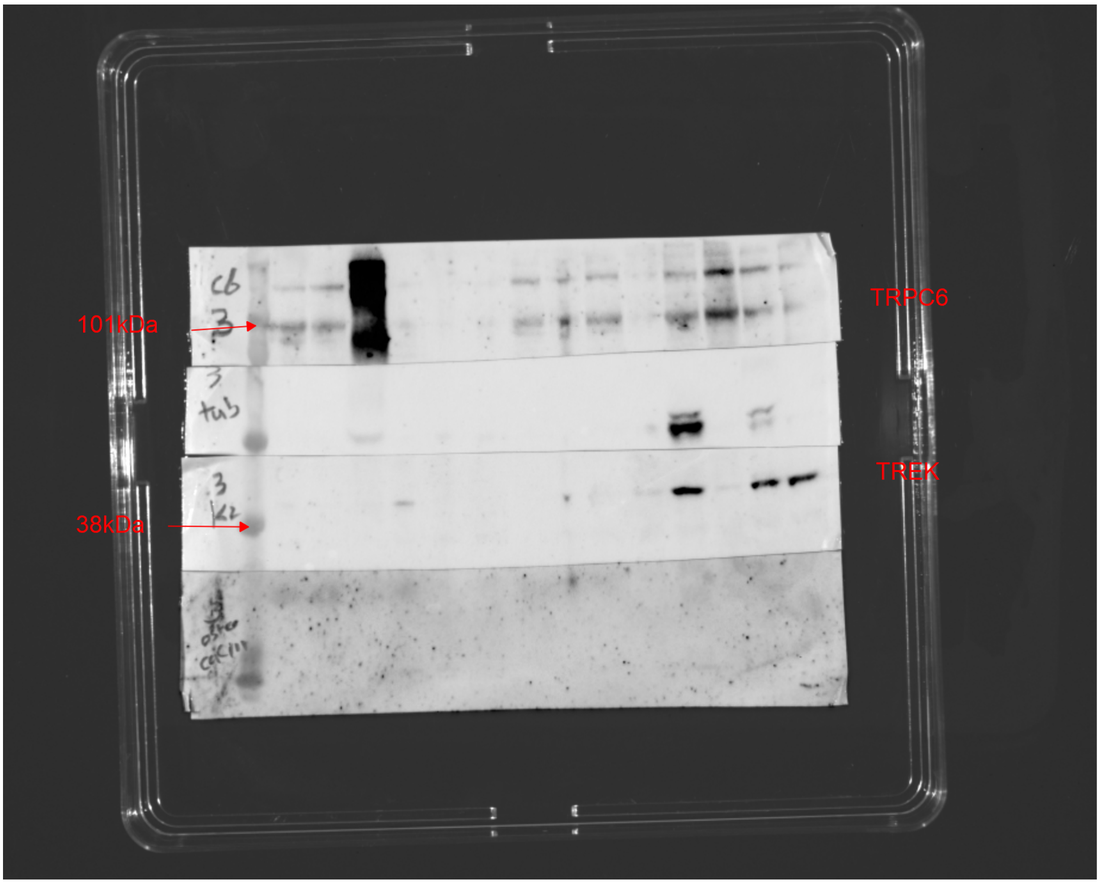

Figure 3
TRPM4

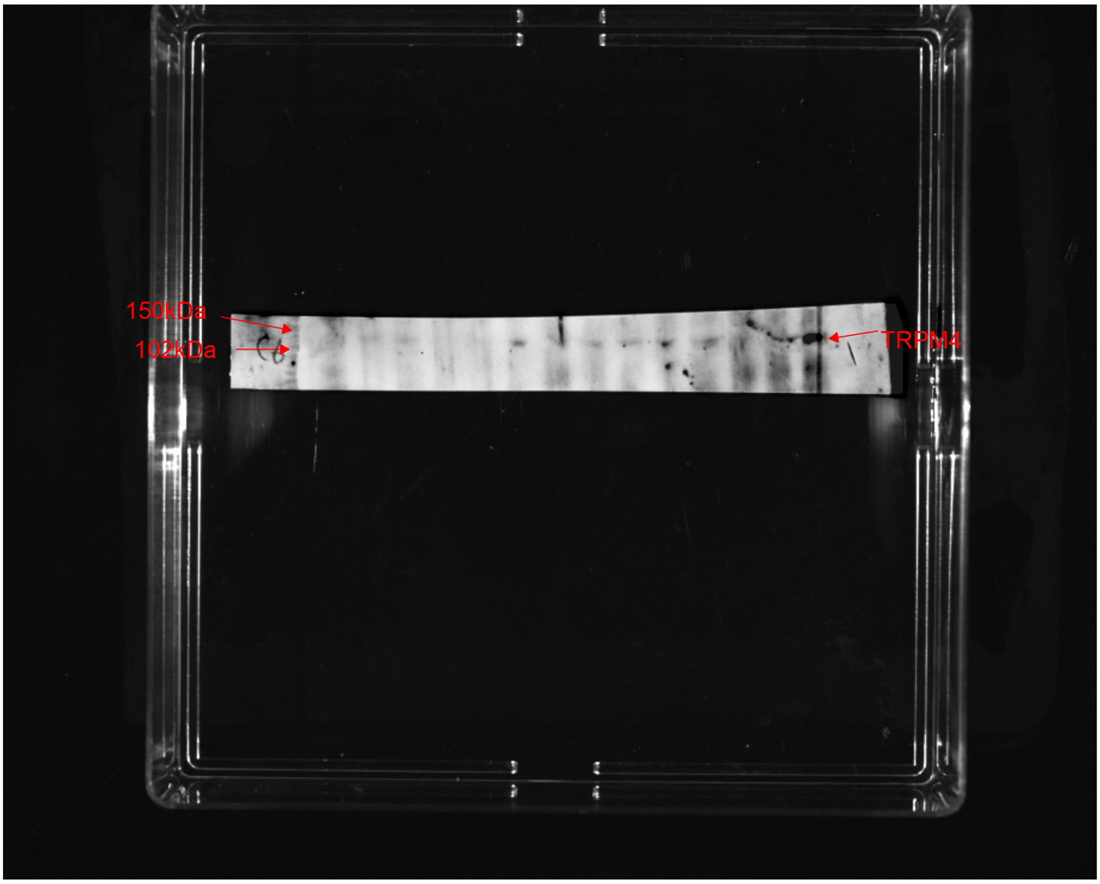

Figure 3
TRPV4

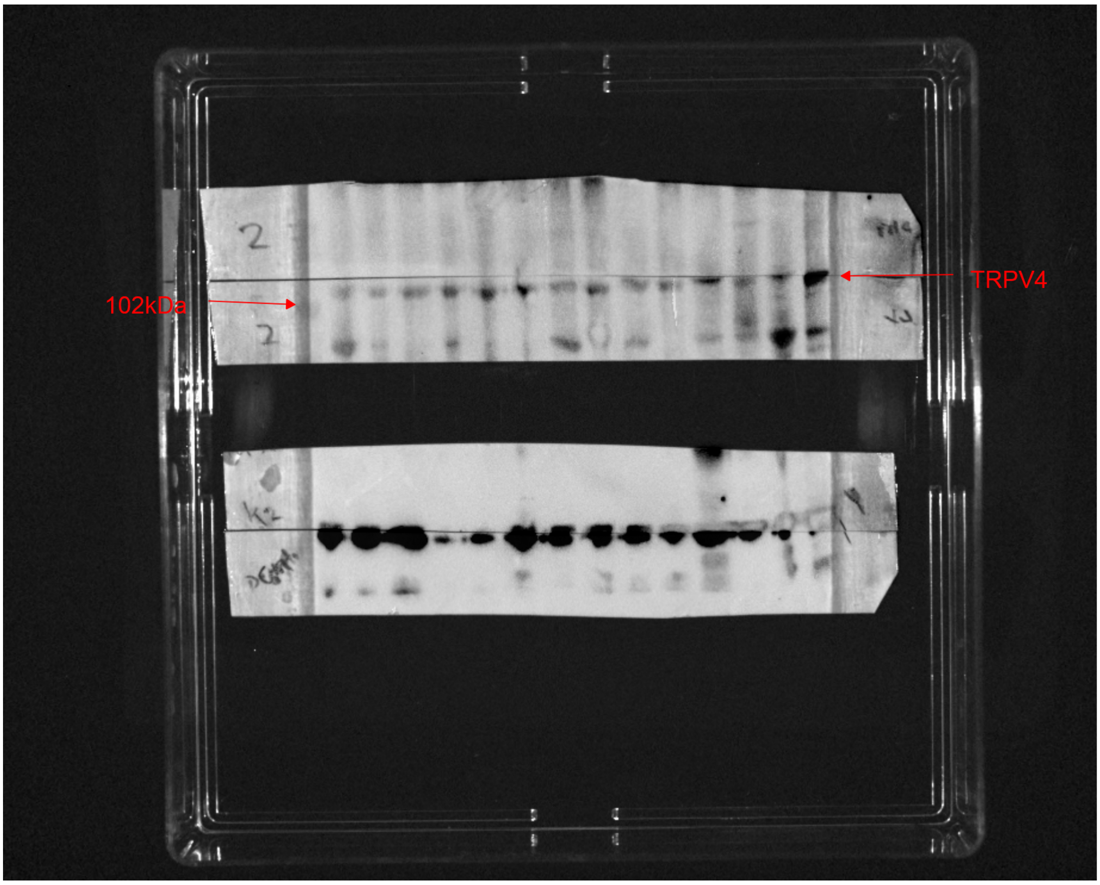

Figure 3
RUNX2

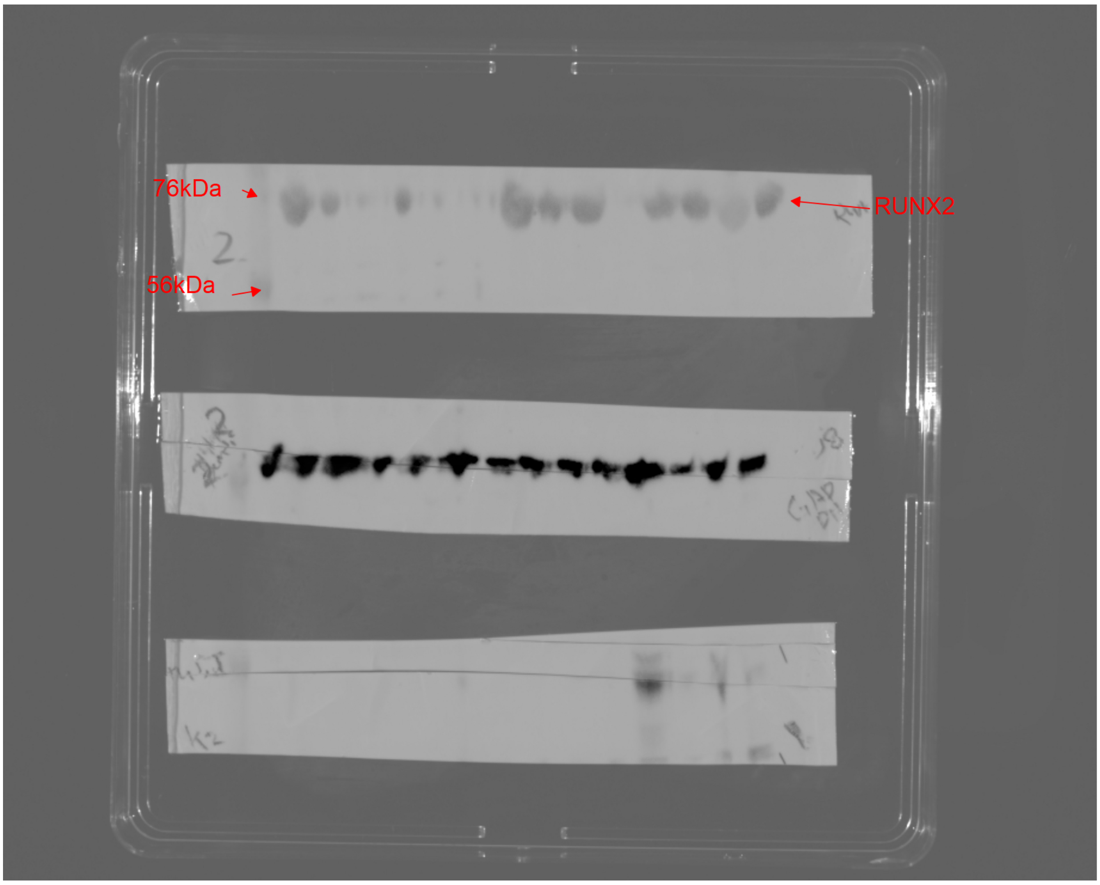

Figure 3
Osteopontin

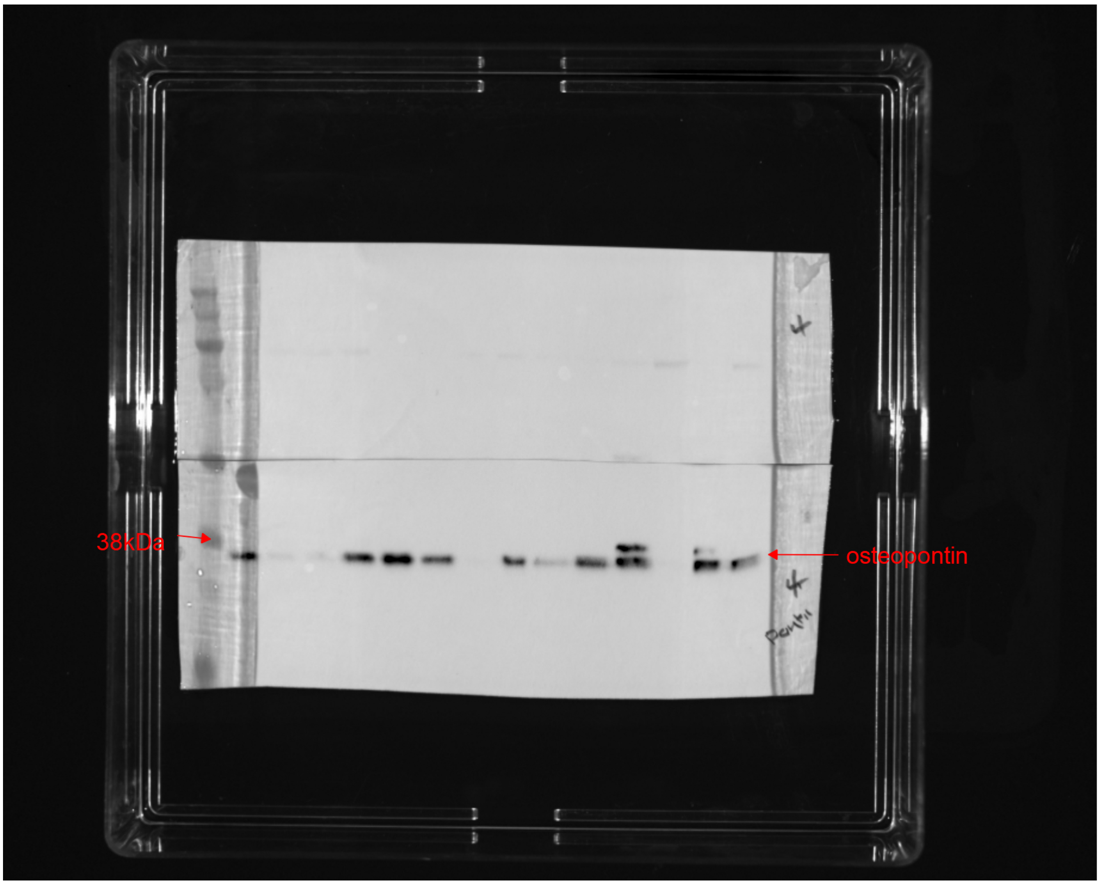

Figure 3
GAPDH

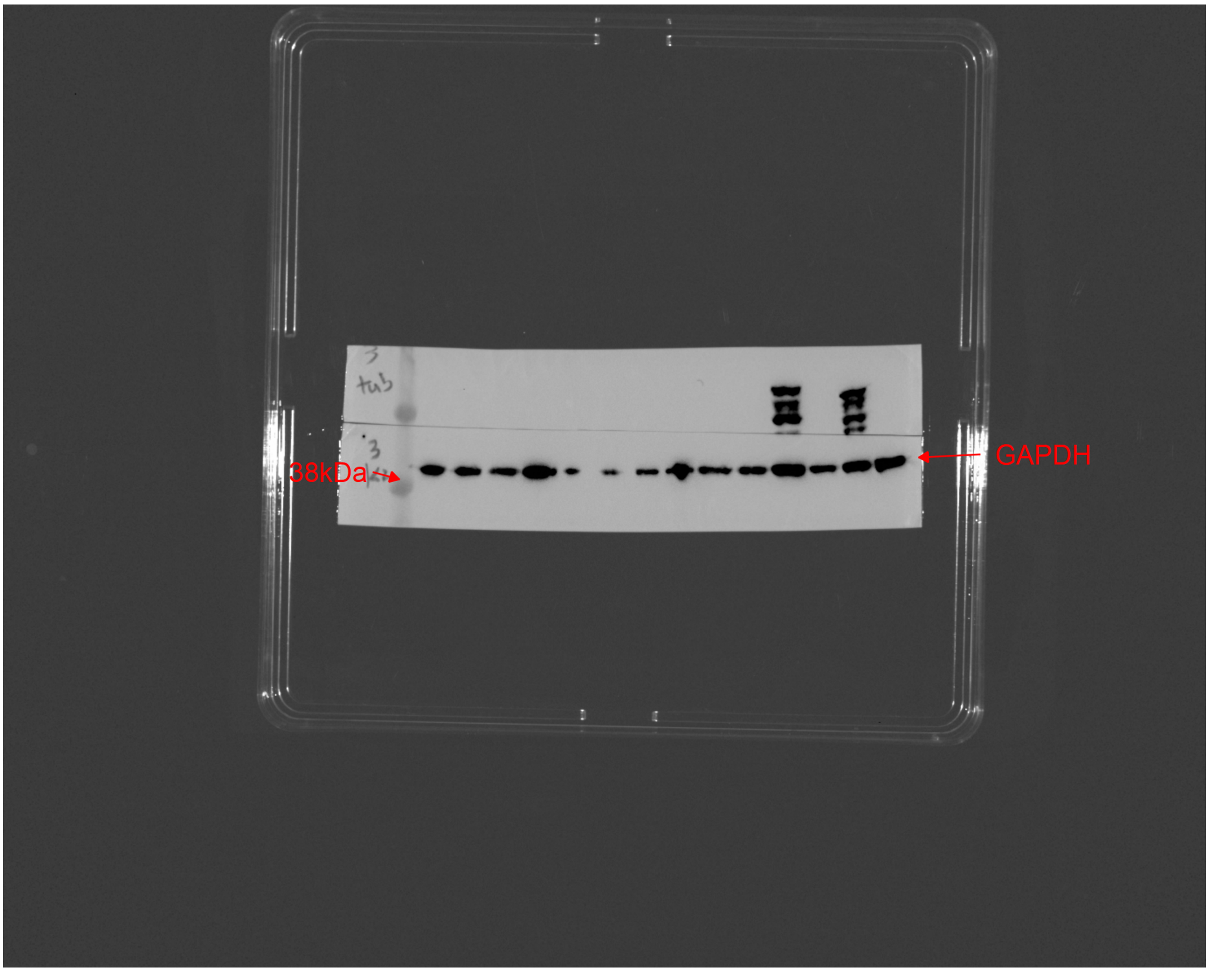

Figure S1
TRPM4, TRPV4, TREK-1 (KCNK2), T-Tubulin

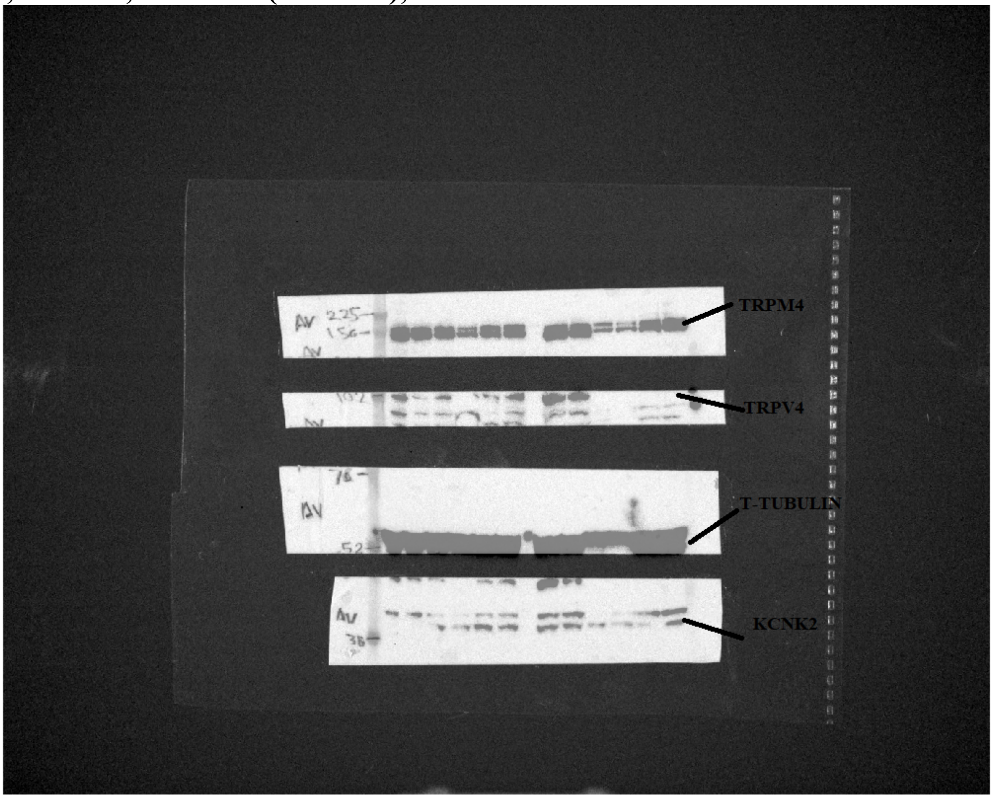

S1 Figure
alpha-SMA

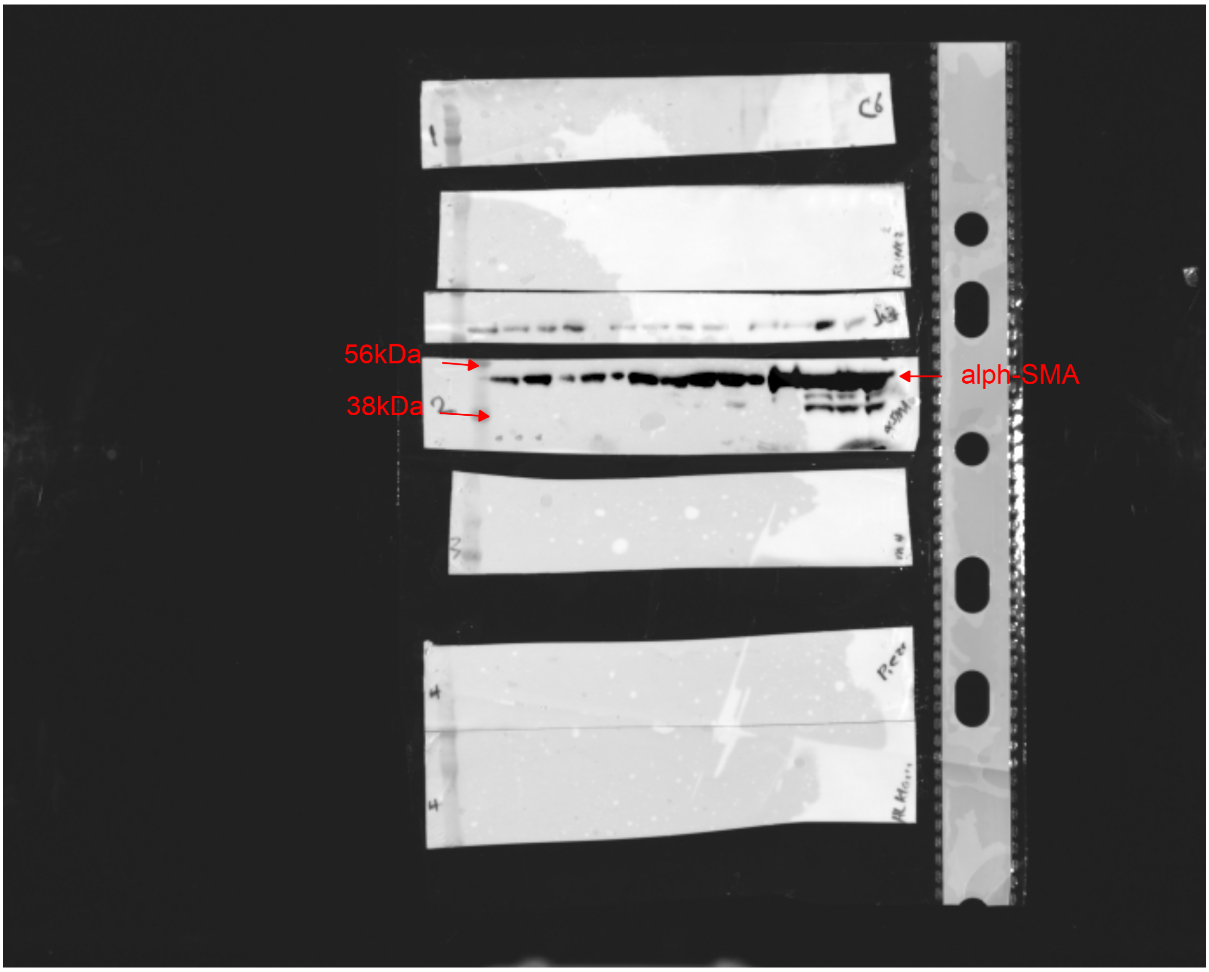

Figure S2
Calponin & SMM-HC

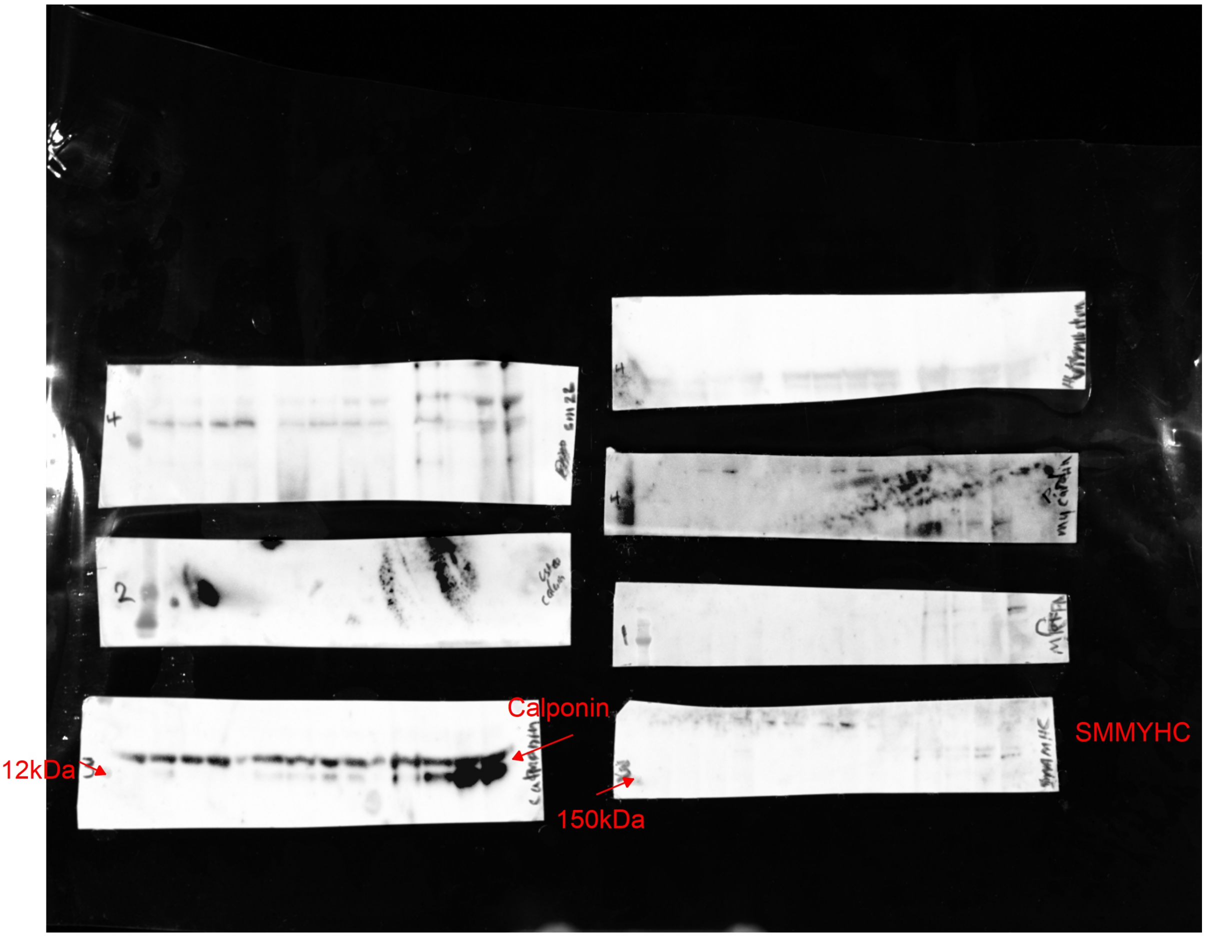

Figure S2
GAPDH

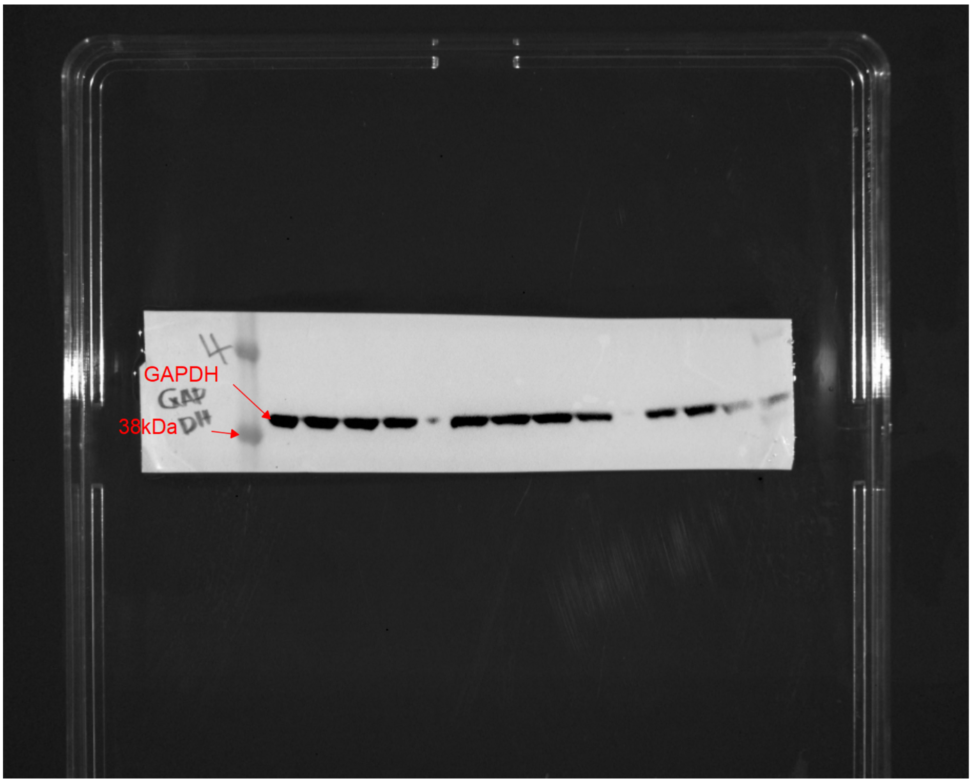

Figure S2
MRTFA

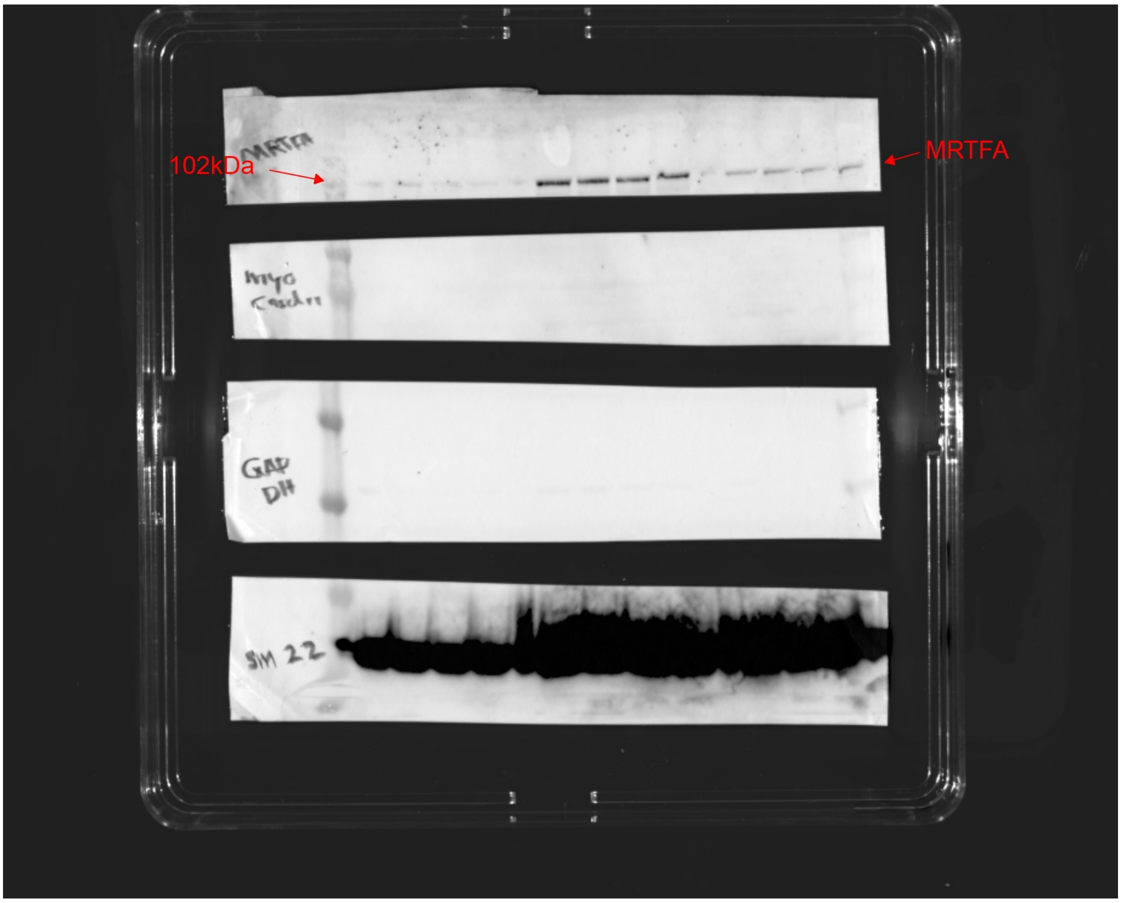

Figure S2
Myocardin

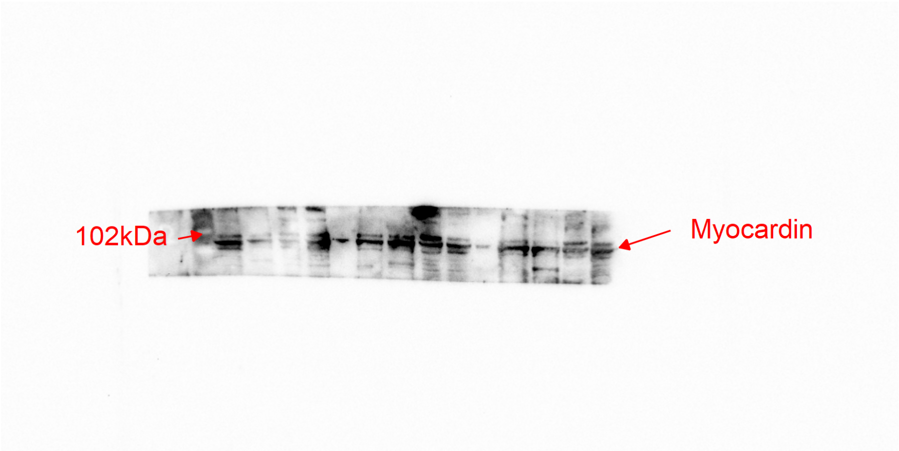

Figure S2
RUNX2

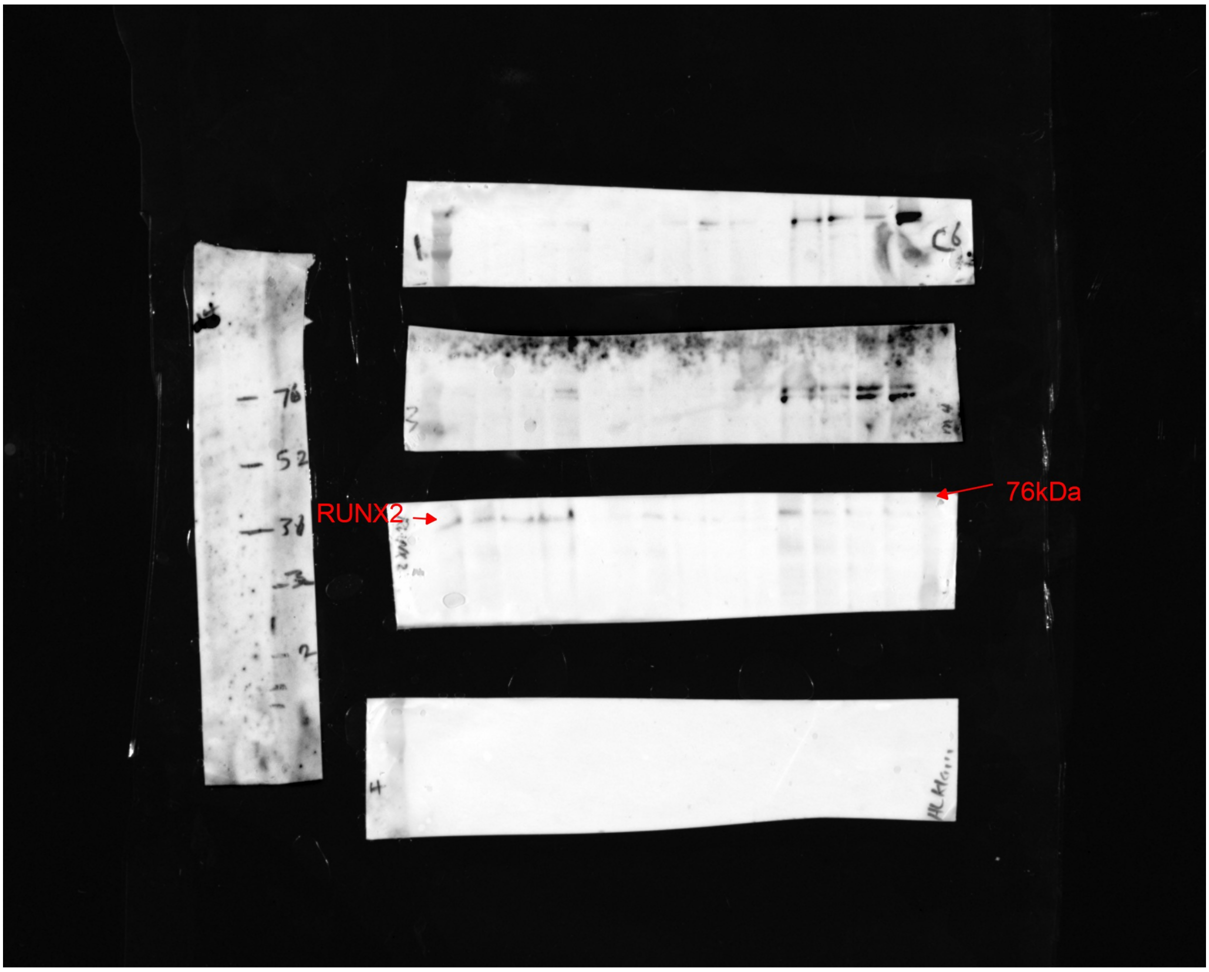

Figure S2
SM22

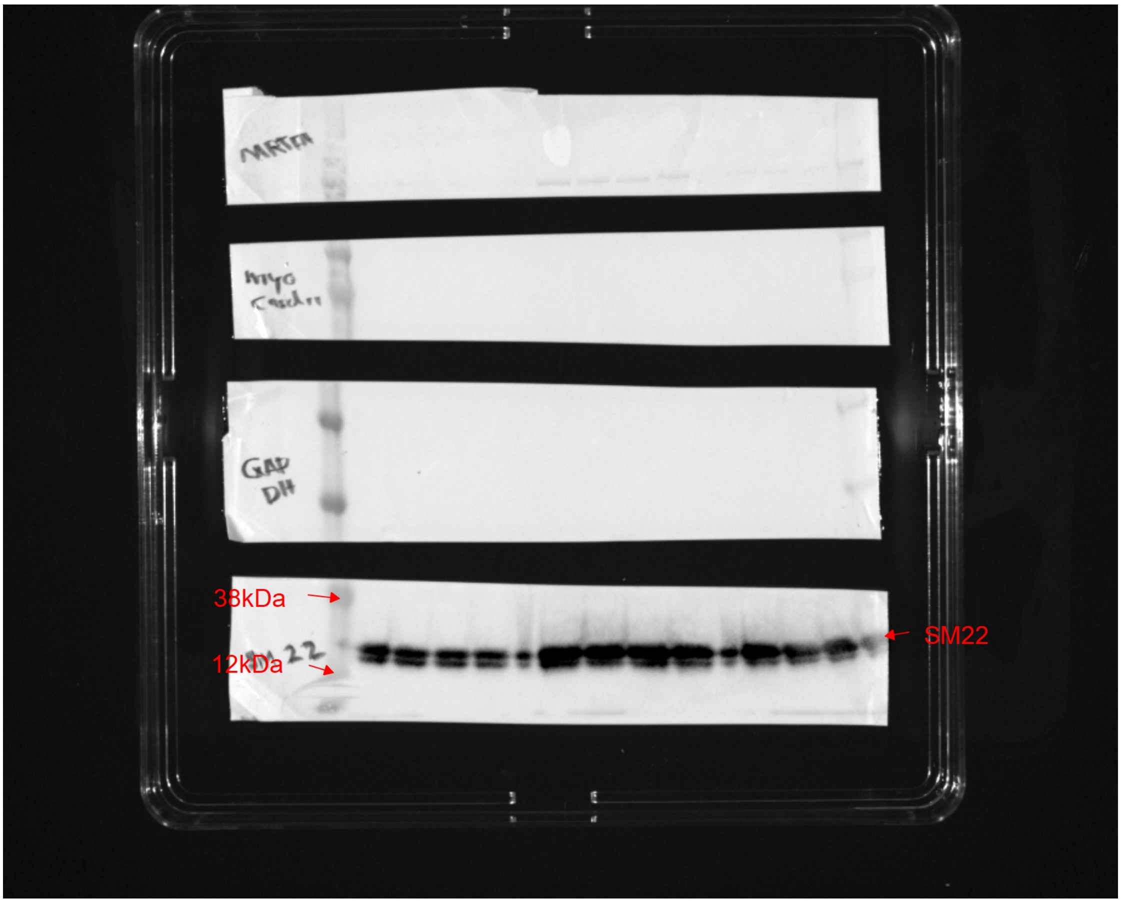

Figure S2
Vimentin

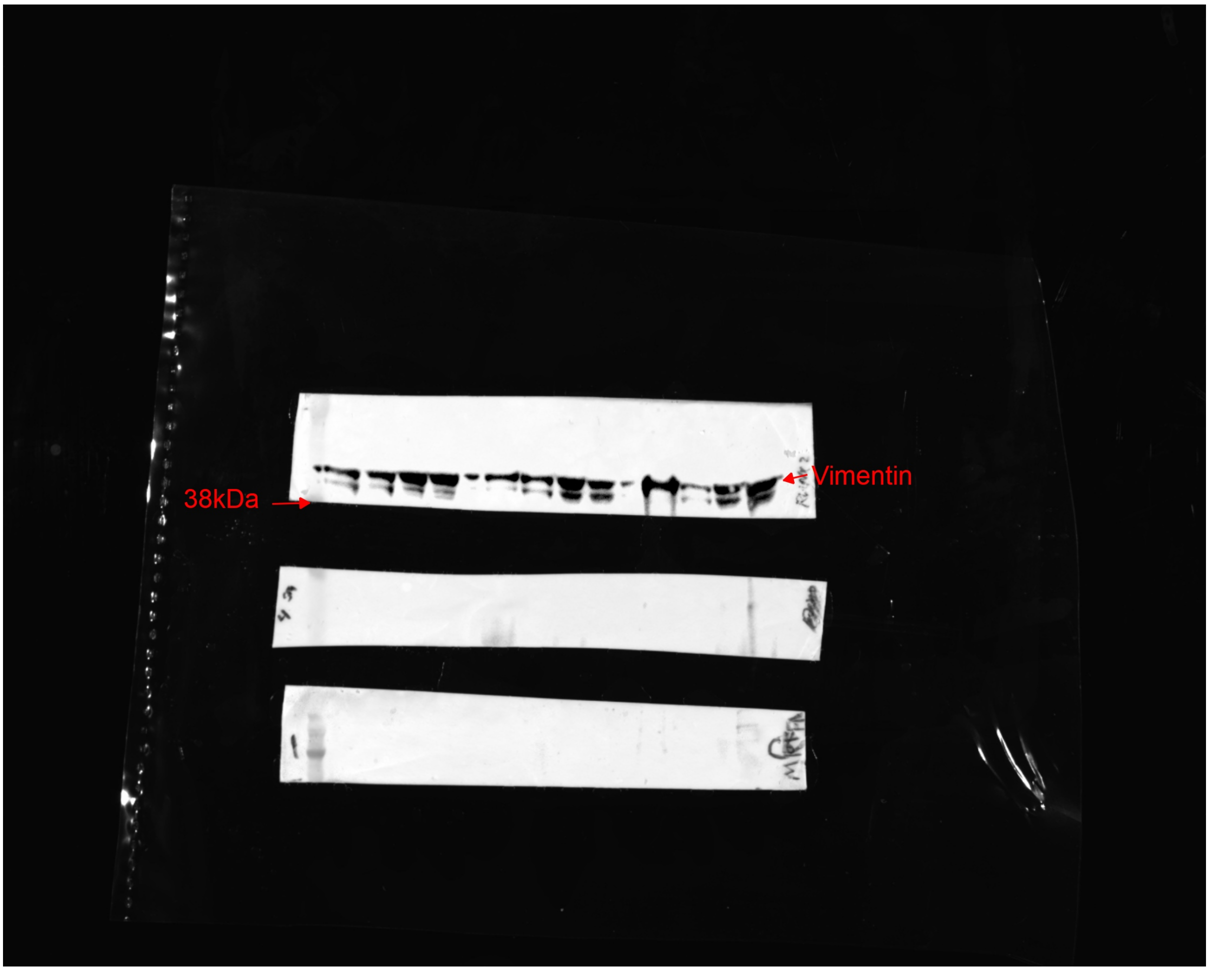

Supplement: S1 Raw images — (ZIP) [file pone.0240532.s004.zip › PONE-D-20-10990 Western Blots Fig2_Fig3_FigS1_FigS2.pdf]
